# Analysing animal social network dynamics: the potential of stochastic actor‐oriented models

**DOI:** 10.1111/1365-2656.12630

**Published:** 2017-02-01

**Authors:** David N. Fisher, Amiyaal Ilany, Matthew J. Silk, Tom Tregenza

**Affiliations:** ^1^ Centre for Ecology and Conservation University of Exeter Penryn Cornwall TR10 9FE UK; ^2^ Department of Integrative Biology University of Guelph Guelph ON N1G 2W1 Canada; ^3^ The Mina and Everard Goodman Faculty of Life Sciences Bar‐Ilan University Ramat‐Gan 5290002 Israel; ^4^ Environment and Sustainability Institute University of Exeter Penryn Cornwall TR10 9FE UK

**Keywords:** animal communities, dynamics, individual‐based models, network‐based diffusion analysis, social networks, transmission

## Abstract

1. Animals are embedded in dynamically changing networks of relationships with conspecifics. These dynamic networks are fundamental aspects of their environment, creating selection on behaviours and other traits. However, most social network‐based approaches in ecology are constrained to considering networks as static, despite several calls for such analyses to become more dynamic.
2. There are a number of statistical analyses developed in the social sciences that are increasingly being applied to animal networks, of which stochastic actor‐oriented models (SAOMs) are a principal example. SAOMs are a class of individual‐based models designed to model transitions in networks between discrete time points, as influenced by network structure and covariates. It is not clear, however, how useful such techniques are to ecologists, and whether they are suited to animal social networks.
3. We review the recent applications of SAOMs to animal networks, outlining findings and assessing the strengths and weaknesses of SAOMs when applied to animal rather than human networks. We go on to highlight the types of ecological and evolutionary processes that SAOMs can be used to study.
4. SAOMs can include effects and covariates for individuals, dyads and populations, which can be constant or variable. This allows for the examination of a wide range of questions of interest to ecologists. However, high‐resolution data are required, meaning SAOMs will not be useable in all study systems. It remains unclear how robust SAOMs are to missing data and uncertainty around social relationships.
5. Ultimately, we encourage the careful application of SAOMs in appropriate systems, with dynamic network analyses likely to prove highly informative. Researchers can then extend the basic method to tackle a range of existing questions in ecology and explore novel lines of questioning.

## Graphical abstract

As a highly social species, we are fascinated by our changing social relationships and those of animals. The dynamic analysis of social networks is regularly called for but infrequently delivered. The authors review a dynamic network analysis technique that can be used to relate social relationships to ecological and evolutionary processes.

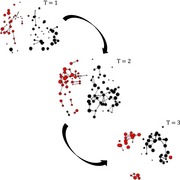

## Introduction

### Social networks in ecology

Animals compete, cooperate and reproduce with conspecifics, and so are engaged in a network of social interactions. These networks represent the social environment of individuals, which influences various evolutionary and ecological processes (Proulx, Promislow & Phillips 2005; Bascompte 2007; Kurvers *et al*. 2014). By simultaneously considering both the traits of the individuals in these networks and their patterns of interactions, networks have been used to study diverse subject areas, such as disease epidemiology and individuality (Weber *et al*. 2013), and the dynamics of group formation (Wilson *et al*. 2014). The importance of links between individual variation and group‐level processes is increasingly appreciated (Farine, Montiglio & Spiegel 2015), and networks are especially useful as a tool to quantify the social environment to which animals are presumed to be adapted. For instance, by quantifying an individual's social network we gain insights into the social information available to it (Aplin *et al*. 2012; Atton *et al*. 2012; Farine *et al*. 2015), the diseases it is exposed to (Hamede *et al*. 2009; Bull, Godfrey & Gordon 2012), the intensity of local competition it experiences (Oh & Badyaev 2010; Formica *et al*. 2011; Fisher, Rodríguez‐Muñoz & Tregenza 2016a) and the strength of its cooperative relationships (Voelkl & Kasper 2009; Apicella *et al*. 2012).

Typically, these networks of relationships are analysed as being static, i.e. a network is built that summarises a period of time, and this network is related to the processes of interest. However, this ignores the fact that individuals may change their interaction patterns over time (Blonder & Dornhaus 2011; Blonder *et al*. 2012). If a relationship between two flexible traits exists (e.g. social connectedness and individual dominance) change in one could drive change in the other, but it is difficult to tease apart which trait drives this relationship when only observing the product. This is true of many processes; for instance, if infected individuals show different levels of behaviour, are they infected because of their behaviour or did the infection change their behaviour or that of those around them? Without an experiment, inference of causality is difficult, but strong evidence can be provided where a process or behaviour is observed to consistently happen before, and lead to a change in, another process or behaviour. This is beyond the reach of static network analyses as it requires time‐ordering to be incorporated into the analyses (Blonder *et al*. 2012; Pinter‐Wollman *et al*. 2013). By modelling change in a network over time, it is possible to identify not only how social and non‐social processes drive each other (Burk, Steglich & Snijders 2007) but also what processes govern the development of network structure (Kossinets & Watts 2006). Furthermore, transmission dynamics, such as the spread of information or disease across a population, can be examined, allowing us to identify factors important for the contraction and transmission of information or disease (Weber *et al*. 2013; Van der Waal *et al*. 2014; Adelman *et al*. 2015; Aplin *et al*. 2015a).

Despite the evident potential in the dynamic network analysis approach, applications in ecology remain relatively limited (but see Blonder & Dornhaus 2011; Jeanson 2012; Wilson *et al*. 2014; Ilany, Booms & Holekamp 2015; Aplin *et al*. 2015a; Borgeaud *et al*. 2016; Pasquaretta *et al*. 2016). Recent calls for the implementation of dynamic network analyses (e.g. Pinter‐Wollman *et al*. 2013; Croft, Darden & Wey 2016) provided theoretical impetus for the use of dynamic networks, but little discussion of appropriate analytical techniques. Furthermore, contemporary introductions to social network analysis for ecologists state that ‘temporal dynamics represent a significant analytical challenge’ and that tools developed by computer scientists ‘are not realistic for many animal social networks’ (Farine & Whitehead 2015). This indicates that we require more accessible methods. Here, we review recent applications of a method for the dynamic analysis of networks: stochastic actor‐oriented models (SAOMs). In the Supporting Information, we provide a practical guide outlining the data requirements, the process of model fitting and how to interpret the results. We also provide a full worked‐through example, complete with an annotated R script and a data set, to allow readers to implement a SAOM.

### The stochastic actor‐oriented model

SAOMs are a class of individual‐based models characterising the behaviour of each actor (individual) in the system, rather than calculating an average effect over a population. The latter approach can be problematic if even small nonlinear dynamics occur (Lehmann 2009). Additionally, linear‐modelling based approaches are often inappropriate for network‐based analyses, as the assumption of independence of residuals is clearly violated when individuals are embedded in an entire network of connections (Croft, James & Krause 2008; Whitehead 2008; Croft *et al*. 2011; Snijders 2011). It is therefore preferable to use a statistical tool specifically designed to model relationships between individuals, and the non‐independence this implies. For a more detailed mathematical description of SAOMs, we refer readers to the RSiena user manual (Ripley *et al*. 2015). Alongside our guide in the Supporting Information, further information on data requirements, model fitting and statistical inference is also available in previous papers that have used SAOMs in animals (e.g. Ilany, Booms & Holekamp 2015; Borgeaud *et al*. 2016; Pasquaretta *et al*. 2016). We will mainly discuss the application of SAOMs as implemented through the program SIENA (Steglich, Snijders & West 2006; Snijders, van de Bunt & Steglich 2010) and the R package RSiena (Ripley *et al*. 2015) as this program allows the full breadth of effects we describe to be implemented and comes with a large body of examples, R code and user guides (see: http://www.stats.ox.ac.uk/~snijders/siena/ for these and http://r-forge.r-project.org/R/?group_id=461 for the package itself).

SAOMs model gradual change in the network and traits of the individuals across discrete time points using hidden Markov models. Individuals may possess consistent positions in their social networks (Blumstein, Petelle & Wey 2013; Brent *et al*. 2013; Jacoby *et al*. 2014; Aplin *et al*. 2015b; Formica *et al*. 2016), and the networks themselves have been shown to be quite consistent across contexts (Dey *et al*. 2015; Firth & Sheldon 2015, 2016), time (Dey *et al*. 2013; Shizuka *et al*. 2014; Ilany, Booms & Holekamp 2015) and even generations (Fisher, Rodríguez‐Muñoz & Tregenza 2016b), so gradual change may be reasonably expected. Individuals are recorded as associating or not at each time point, i.e. the networks are binary. The duration of each time period will be determined by the study system, the questions and processes being investigated and the resolution of the data available, and/or through pilot‐analyses to determine the most appropriate resolution (e.g. Pasquaretta *et al*. 2016). Some studies of human associations have used up to yearly censuses (e.g. Steglich, Snijders & West 2006), although shorter time frames are more likely to be used for animal social networks (for instance we used 8 days for the example in our Supporting Information).

Being modelled as a Markov chain means that information about the past is not included by default and is assumed to not bring any additional predictive power (Burk, Steglich & Snijders 2007; Snijders, van de Bunt & Steglich 2010). While this may initially seem an oversight, it should be noted that SAOMs model states, e.g. ‘X and Y are currently connected’, rather than events, e.g. ‘X interacted with Y’ (Snijders, van de Bunt & Steglich 2010), so historical information on long‐term social associates is included in present information. However, even if researchers have recorded events rather than states, these data can be used in a SAOM. Events can be aggregated to infer states (Snijders, van de Bunt & Steglich 2010), e.g. ‘X and Y were grooming each other 4 days out of 7 this week, suggesting they are socially affiliated’. Between each time point, it is assumed that individuals optimise their position in the network according to a utility function, with this function determined by their links with others in the network and the links between these others, short‐term preferences and unknown tendencies (modelled as residual/random deviance; Burk, Steglich & Snijders 2007). The process of change between each time‐step consists of the objective function and the rate function. The objective function determines the manner of the change occurring, e.g. which individuals form social relationships, while the rate function models the speed of change of relationships, e.g. if individuals slowly form bonds that last a long time, or rapidly form short‐term relationships. Each of these functions can be influenced by covariates related to individuals or the environment. Two further assumptions worth highlighting in our description of the modelling framework are that (i) it is assumed that each individual controls it outgoing interactions, and (ii) that each individual has complete information about the network. However, neither of these assumptions is overly restrictive. For the former, in directed networks, outgoing interactions are typically defined as those the individual initiates, while for undirected networks, SAOMs allow multiple definitions of interaction that can correspond to the studied system, for example a relationship formed either by the actions of a single individual or the mutual agreement of both individuals in a dyad (Ripley *et al*. 2015). Regarding complete knowledge of the network, individuals typically only need limited information to act as they do, as the local network is typically the main driver of network change (Snijders, van de Bunt & Steglich 2010). Therefore, findings are generally robust to minor violations of this assumption.

**Figure 1:**
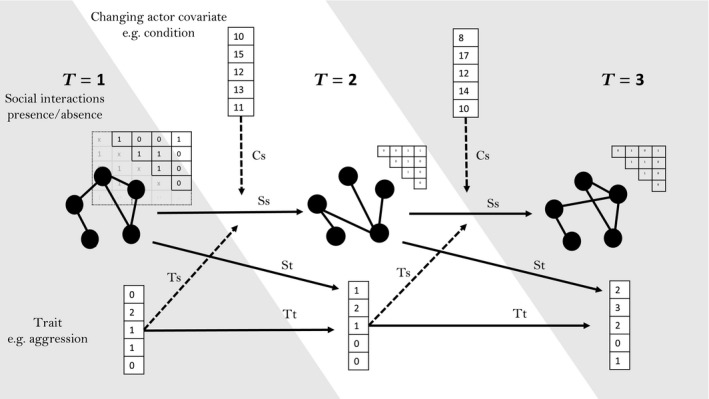
Pictorial representation of a SAOM, to illustrate the kind of effects that can be modelled. Note that our recommendations on network size still apply (see Supporting Information). Here there are three time periods, where five individuals change (or not) their social associations over time. Simultaneously, there is another dependant variable (a trait value, e.g. aggression) changing across each of the three time periods. Processes depicted model effects of: the social structure at one time point depending on the social structure at previous time points (lines labelled ‘Ss’); social structure influencing the value of traits at the next time point (lines labelled ‘St’); the trait at one time point influencing the trait at the next time point (lines labelled ‘Tt’); the trait influencing how the social structure changes from one time point to the next (lines labelled ‘Ts’) and some changing actor variable (e.g. condition) influencing the social structure change from one time point to the next (lines labelled ‘Cs’). Here the network is undirected/symmetrical, so only the above‐diagonal of the association matrices are shown at time points two and three, but full association matrices would be entered as data for all.

SAOMs model network change in a series of network snapshots, including each member of the population and their observed social associations at each time point. Change in the network can be modelled in response to connections in the network and in response to a range of covariate types. Furthermore, SAOMs can include traits of individuals as response variables, which may be modelled to vary due to covariates or covary with social relationships. We describe each of these below. Figure 1 provides a pictorial representation of a SAOM, indicating the breadth of effects that can be specified. Descriptions of some of the network and trait processes that SAOMs can model of interest to ecologists are provided in Table 1, with some implemented in our worked example in the Supporting Information. A more complete list is available in the RSiena manual (Ripley *et al*. 2015).

**Table 1:** A list of possible effects of particular interest to ecologists that can be modelled with SAOMs in the SIENA software. In general, a positive value for the effect indicates the process outlined is occurring, but if otherwise this will be described. Effect type indicates whether the effect is a structural term, a covariate influencing the network, or if it involves the relationship between tie formation and the change in a trait, and whether the effect is relevant for undirected and/or directed networks. ‘Ego’ refers to the individual who is initiating the interaction, ‘alter’ to the receiver of the interaction

Effect name	Effect type	Description of effect	Behavioural process
Ego/alter effects on tie formation	Covariate, directed and undirected	Traits of the individual on the ties it sends/receives	Traits of individuals, e.g. their sex or age influencing the likelihood to form ties
Ego/alter effects on rate	Covariate, directed and undirected	Traits of an individual on rate of change of relationships	Individuals of different sex, age or personality forming or dissolving ties at different rates
Ego‐alter trait interactions	Covariate, directed and undirected	Properties of both individuals on the chance of tie formation between them	Positive: ties form within classes/homophily, e.g. intra‐sex aggression Negative: ties form between classes, e.g. producer‐scrounger
Outdegree	Structural term, directed	Number of existing associations of an individual on its tendency to form new associations	Positive: Social behavioural types, e.g. consistently social or non‐social individuals Negative: optimising group size
Popularity/indegree	Structural term, directed and undirected	Tendency for individual to associate with others who already have a large number of associates	Attractive/susceptible phenotypes for affiliative/aggressive interactions
Triadic closure	Structural term, directed and undirected	Tendency of individuals to associate with ‘friends of friends’	Coalition/clique formation
Reciprocity	Structural term, directed	Individuals repeat interactions with those that interact with them	Preferred associations, tit‐for‐tat cooperation
Balance	Structural term, directed and undirected	Tendency to have/lack the same ties as another associate	Partner choice copying, community formation
Three cycles	Structural term, directed	Directed social interactions, e.g. grooming or aggression, from X to Y, Y to Z and Z to X	Positive: generalised reciprocity Negative: linear dominance hierarchies
Influence	Network‐behaviour co‐dynamic, directed and undirected	Changes in individuals’ traits due to the behaviour of their associates	Social learning and information or disease transmission
Selection	Network‐behaviour co‐dynamic, directed and undirected	Forming ties due to the behaviour of the other individuals	Positive: partner choice based on phenotype Negative: avoidance of aggressive or diseased individuals
Dyadic covariates	Covariate, directed and undirected	Properties of a relationship between two individuals, e.g. distance	Accounting for separation in space, time or degree of genetic relatedness between individuals
Degree on behaviour	Network‐behaviour co‐dynamic, directed and undirected	Influence of number of relationships on behaviour	Social behaviour carry‐overs to non‐social contexts, e.g. Winner‐loser effects
Behaviour on degree	Network‐behaviour co‐dynamic, directed and undirected	Influence of behaviour level on formation of new ties	Behavioural carry‐overs to social contexts, e.g. boldness covaries with frequency of aggressive interactions

The SAOM framework can estimate the importance of a variety of structural network processes (e.g. the tendency of individuals to form associations with individuals with whom they already share a mutual associate: ‘triadic closure’) on network change. Modelling these kinds of structural processes allows the researcher to determine how particular aspects of individuals’ social environments, such as the presence of a mutual associate, influence their choice of association partners. Such effects also enable researchers to control for structure in the data or biases generated by the method of data collection (see below).

The inclusion of covariates (at both individual and dyadic levels) enables the assessment of the role of individual traits and other relationships between individuals in influencing network structure. Individual traits can be included as constant actor covariates over the time period (e.g. sex) and, equivalently, fixed dyadic traits (e.g. relatedness) can be included as constant dyadic covariates. Additionally, dynamic covariates can also be included, either for changing individual traits (e.g. body condition), changing environmental conditions (e.g. rainfall) or changing dyadic covariates (e.g. spatial proximity). Interactions between network effects and covariates can also be specified. For instance, in some social systems it might be hypothesised that males are more likely to form coalitions than females. One would then specify an interaction between sex and triadic closure, and evaluate its importance.

Finally, behaviours or traits, as long as they change in a similar time frame to social relationships, can be considered as response variables alongside network change, allowing their change to be directly modelled alongside the change in social relationships. If the trait does not vary at a similar temporal scale as the variation in the social network, then their relationship cannot be assessed, as SAOMs model network dynamics and trait changes on the same temporal scale. By modelling how an individual's trait changes over time, affected by the trait values of those it interacts with, one can also model the spread of information, a cultural trait or disease across a population (see Example research area 1 below).

### Previous applications of SAOMs

SAOMs were initially developed in the social sciences, and have been used extensively to study human behaviour. Example research questions include how music preferences and drug taking habits develop within and among friendship groups (Steglich, Snijders & West 2006) and how unethical behaviour can spread within organisations (Zuber 2014), see Wölfer, Faber & Hewstone (2015) for a review. Such questions have clear analogies for non‐human animal behaviour (such as the spread of a novel foraging technique through a group; Boogert *et al*. 2008; Allen *et al*. 2013; Aplin *et al*. 2015a). To date however, there have been only limited applications of SAOMs by those investigating animal interactions, although this has started to change in the last few years.

Jones (2011) investigated patterns of interactions in farmed salmon *Salmo salar*, and found that fish were either consistent givers or receivers of aggression, suggesting social personality types (Krause, James & Croft 2010; Wilson *et al*. 2012; Aplin *et al*. 2015b). More recently, Ilany, Booms & Holekamp (2015) investigated the long‐term dynamics of spotted hyena *Crocuta crocuta* social networks. Some of their key findings were that structural constraints, individual's traits and environmental conditions all shape network dynamics, and that female hyenas are more flexible in their social bonding tendencies, possibly reflecting their dominance in hyena groups (Ilany, Booms & Holekamp 2015). SAOMs have also been used to investigate social information transmission in *Drosophila melanogaster* (Pasquaretta *et al*. 2016), showing that uninformed flies tend to change social contacts faster. Boucherie *et al*. (2016) used SAOMs to explore changes in relationships in captive rooks *Corvus frugilegus*. Rooks preferentially interacted with paired congeners and were more likely to develop a relationship with connections of a social partner. They also found that sex had no significant effect on social dynamics. Finally, Borgeaud *et al*. (2016) investigated the dynamics of multiple social groups of vervet monkeys *Chlorocebus pygerythrus*, and found that some processes (e.g. triadic closure) were key to all groups, while others varied in their importance.

These studies provide fundamental insights into how and why animal groups from a range of taxa possess their observed structure, in particular highlighting the varying importance sex plays in different social systems and the importance of accounting for topological network effects on social relationships. Each of these studies had access to a large enough population of animals that were reliably individually recognisable, where social relationships could be clearly defined and when (relatively) complete covariate data were available. The same will need to be true for other studies that attempt to utilise SAOMs. In fact, the data requirements may be even more severe to fully exploit SAOMs. Specifically, as we have highlighted in the preceding section, SAOMs can model multiple traits changing over time, not just social relationships, and the individual or environmental covariates that affect them. Furthermore, it is possible to model the effect of greater network structure (beyond immediate connections) on the formation of new ties. These elements will require high‐resolution data on the changing social relationships and traits of a whole population of individuals at multiple time points, which will prove a challenge for the application of SAOMs to typical data sets in ecology.

In summary, this approach allows researchers to model the combination of the change in a trait over time, social relationships, group behaviour and transmission dynamics. Having outlined the opportunities that exist when using SAOMs (above and Table 1), we now describe some of the challenges that still need to be addressed. The data requirements for a SAOM will be the major challenge for ecologists wishing to use this method (see Supporting Information for technical detail). We then provide examples of research areas SAOMs seem particularly suited to tackle and discuss the modelling and simulation work needed to establish the performance of SAOMs with data sets more typical of those in ecology.

## Challenges when using SAOMs to study animal networks

Above we highlighted the diversity of effects that SAOMs can model, and the recent applications in a range of organisms. This should make clear the opportunities to be exploited when using SAOMs to investigate dynamic network processes. However, as touched upon above, there are significant challenges when applying SAOMs to animal network data. Five principal concerns are that: (i) SAOMs only model relationships as existing or not, i.e. they cannot accept relationships of different strengths, (ii) they are not designed to deal with situations where there is uncertainty surrounding network edges, (iii) missing individuals (i.e. those not identifiable in the population) or interactions can lead to problems with estimation, (iv) methods of data collection may bias the network and (v) adequate controls for spatial proximity for non‐social reasons are required.

### Binary networks and the inclusion of interaction strength

SAOMs are designed to study change in the presence and absence of social relationships, i.e. binary networks. Reducing weighted networks to binary descriptions can have major implications in animal social network analysis, for instance if weak ties are important for processes such as information transmission (Granovetter 1973) and may result in incorrect network metrics (Franks, Ruxton & James 2009; Farine 2014). As SAOMs split the data collected into distinct time periods, some information on repeated associations is retained in the form of relationships being present in multiple time periods rather than a single interaction with a weight. Furthermore, through the use of ‘ordered’ networks, RSiena can model relationships from a small range of different strengths. Essentially, a binary network of ‘strong’ associations among a population is entered alongside another binary network of ‘weak or stronger’ associations (theoretically three or more levels of association strength could be used, although the tractability of the subsequent model may limit such extensions). The SAOM then estimates what influences weak association formation and dissolution, and what predicts the transitions between weak and strong associations. This avoids some of the problems associated with ‘filtering’, where ties below a certain threshold are removed, as ties can be represented as belonging to a small set of different strengths. Determining these association strengths still requires a degree of thresholding however, so the problems mentioned above are still present to some degree. Therefore, SAOMs are likely to be most appropriate when the key biological implications of the interaction depends on whether it happened or not (e.g. sharing a nest or roost, creating the opportunity for direct transmission of a parasite), with limited additional information provided by assigning a range of weights to relationships. Methods to analyse networks with edge weights drawn from a greater range through (the related) exponential random graph models are being developed (Krivitsky 2012), so in the future, SAOMs may be able to include such information.

### Edge uncertainty

Animal networks typically contain greater uncertainty than human networks, as we must infer unobservable social states from observable behaviours. Ideally, we would use SAOMs when limited inference of unobserved social relationships is required (e.g. when two primates are observed to groom one another). However, if association‐based methods are used (i.e. social relationships are inferred from repeated spatio‐temporal co‐occurrence, e.g. Sundaresan *et al*. 2007; Shorrocks & Croft 2009; Aplin *et al*. 2012; Allen *et al*. 2013; Ilany, Booms & Holekamp 2015) during network construction then a high level of confidence that these data represent true states of association is required. For some study systems and some methods, this may require a large number of observations (Lusseau, Whitehead & Gero 2008; Franks, Ruxton & James 2009; Farine & Strandburg‐Peshkin 2015). This however does not preclude their use (e.g. Ilany, Booms & Holekamp 2015) as long as the inference of associations is a confident one. To increase confidence in the results, one can use multiple thresholds to construct binary networks, and then to model network dynamics using each data set to evaluate the sensitivity of the results to a given threshold. Assuming any major biases are accounted for, transitions from one network to the next should still approximate real changes in the animals’ social environments. RSiena parameter estimates are provided with standard errors, which will be large if the studied effects are weak or highly variable. As mentioned above, if you cannot reliably infer states of association from your data, we do not recommend the use of SAOMs.

### The impact of missing individuals and interactions

Animal networks typically contain both missing individuals (e.g. when an individual was not observed for the entirety of the study period) and missing edges (e.g. an association between two individuals was missed). Individuals appearing or disappearing from the network during a study can be accounted for in the modelling process. In RSiena, the absence of an association due to missing data can be entered so that the absence of the relationship will not inform parameter estimates (see the Supporting Information and Ripley *et al*. 2015), which we feel is an acceptable solution. However, individuals or edges that are never detected throughout the entire study period represent uncertainty that cannot be addressed in this way. In general, this is a problem that applies across statistical approaches to network analysis rather than to just SAOMs (Kossinets 2006), the consequences of which depend on the proportion of individuals missing and whether they were missing at random or not (Smith & Moody 2013; Silk *et al*. 2015; Smith, Moody & Morgan 2017). Missingness can affect parameter estimates through a reduction in network size, which has knock‐on effects on network parameters, and non‐random missingness related to variables of interest that may bias results (Huisman & Steglich 2008). Hipp *et al*. (2015) found that some parameters in a SAOM are robust no matter how missing data are dealt with, but other parameters are dependent on how missingness is handled. Methods of imputation of this missing data are viable, provided that the method is mindful of the study design (e.g. the ‘Held‐Out Predictive Evaluation’ of Wang *et al*. 2016). Methods that generate uncertainty around observations of animal networks (e.g. Farine & Strandburg‐Peshkin 2015) and simulation studies that explore the effect of missing information on the outcome of analytical approaches (e.g. Silk *et al*. 2015; Smith, Moody & Morgan 2017) will be essential for exploring how robust SAOMs are to the kinds of missing data common in ecological rather than sociological data sets.

### Biases introduced by the method of data collection

The method of data collection may influence the network structure, creating spurious patterns. For example, individuals will be assigned many mutual associations if all individuals in a group are linked when they are observed together (the ‘gambit of the group’; Whitehead & Dufault 1999). This gives strong importance to the effect of triadic closure, as individuals will be connected to all their groupmates as well as to each other (Franks, Ruxton & James 2009). Performing enough censuses or surveys can ameliorate this problem (Franks, Ruxton & James 2009). Furthermore, there are additional features of SAOMs that allow the control of various factors that may bias results if they are known: particular structural network terms, or covariates, can be used to model aspects of the social network that may stem from the method of data collection. For example, when modelling networks constructed using a group‐based approach, the estimate for triadic closure could be considered to be (at least in part) controlling for this effect rather than being a parameter of interest. Additionally, dummy variables that interact with certain effects can be used to control for the confounding effect of a methodological bias, e.g. higher detection rate and so a higher frequency of interactions in certain study areas. This then allows conclusions about hypotheses of interest to be made having accounted for the confounding factors. The type of controlling factor specified will depend on the likely biases a particular method of data collection introduces, provided this is known. If the biases a method introduces are not known, then simulations may need to be performed to determine what the null expectations in the system are, to compare to the observed outcome.

### The effect of spatial structure in networks

Spatial factors can influence the likelihood of two individuals interacting in a wide range of animal networks (Frère *et al*. 2010; Carter *et al*. 2013; Best *et al*. 2014). This raises the possibility that we are studying co‐choice of spatial location rather than choice of social partners, as certain patterns of resource exploitation can create the impression of complex social behaviour (Ramos‐Fernández, Boyer & Gómez 2006). Therefore, we need to account for animals’ space use when assessing their choice of interaction partners. Within a SAOM, this issue can be tackled through the use of dyadic covariates in the model. Shared group membership or a spatial relationship such as the distance between locations at the start of each time point can be entered as a dyadic covariate which accounts for the fact that individuals in the same group or near each other are more likely to interact. This effectively incorporates an appropriate null model into the analysis, as the significance of other parameters in the model is calculated alongside the influence of these spatial control terms. Therefore, preference for associating with (for example) individuals of the opposite sex can be estimated given the degree to which the sexes use the same space. A similar approach is advocated by Whitehead & James (2015), who suggest calculating ‘generalised affiliation indices’ (GAIs) that represent relationships that occur beyond what is expected based on factors such as spatio‐temporal overlap to enter into further network analyses. Such GAIs could be entered into a SAOM, but we recommend using the original association data and the factors that need controlling for within the SAOM. Spiegel *et al*. (2016) have recently developed a modelling strategy to separate spatial proximity from social associations, using randomisations of movement patterns within individuals with particular time periods. The resulting ‘expected’ patterns of social association could then be entered as changing dyadic covariates into a SAOM, as described above. An outstanding problem for these approaches is the degree to which the spatial position of individuals itself represents social behaviour, so should not be ‘accounted for’ and discarded from inferences on social behaviour. Assessing how different controls for spatial location influence our inferences about social behaviour in different systems should inform us on how problematic this issue is.

## Example research area 1: SAOMs and transmission dynamics

A primary interest for those studying epidemiology is how individual behaviour relates to infection at the individual and the population level (Tompkins *et al*. 2011). If a disease is transmitted directly, its spread depends on the social relationships of the entire population, making it a network‐based problem. With a SAOM, being infected or not can be modelled as a dynamically changing trait with multiple levels (e.g. uninfected, infected but dormant, infective). This can then be influenced by (i) individual characteristics (e.g. sex, condition), (ii) network position (e.g. connectedness) and (iii) the characteristics of associates, including their own disease state. This allows the tendency to be infected to be influenced by the infection status of social partners, allowing the spread of a disease across the dynamically changing network to be modelled. The researcher can then explore whether the infection status alters the rate or choice of interactions, or the tendency to be targeted with interactions. As well as modelling disease status as more complicated than infected/uninfected, differences between classes (e.g. sex) in infection or transmission rates can be examined (McDonald *et al*. 2014). Furthermore, the change in infection status could be constrained to becoming infected, with returns to an uninfected state being impossible (Greenan 2015; Ripley *et al*. 2015). A similar framework can be applied to information transmission, modelling the spread of information across a population (Greenan 2015). This has previously been investigated in animals using network‐based diffusion analysis (NBDA; Aplin *et al*. 2012, 2015a; Atton *et al*. 2012; Allen *et al*. 2013; Boogert *et al*. 2014; Farine *et al*. 2015) with Hobaiter *et al*. (2014) extending traditional NBDA to account for the build‐up of relationships over time. However, SAOMs explicitly model both the change in the network and in the trait over time as mutually connected dependant variables. This allows the extent and direction of any causal relationship(s) and the effect of external variables on the change in both networks and the trait to be modelled. This gives different information to NBDA, which may instead be used to estimate the transmission speed across different parts of and the whole of the network.

## Example research area 2: Behavioural types and networks

Social network analyses determine how specifics of individuals’ social environments, as measured by network traits such as degree or betweenness, are related to other aspects of their ecology. This indicates that individual‐level behavioural traits are important for ecological and evolutionary processes. This conclusion has become an established orthodoxy in behavioural ecology (Koolhaas *et al*. 1999; Dall, Houston & McNamara 2004; Réale *et al*. 2007), with within‐population, among‐individual differences in behaviour observed to be widespread (Bell, Hankison & Laskowski 2009), linked to fitness (Smith & Blumstein 2008) and various ecological and evolutionary dynamics (Wolf & Weissing 2012). By modelling both as responses, SAOMs allow the integration of these two branches of individual specific behaviours from social and non‐social domains, studying their codependent change over time. For instance, one could model how the level of risk‐taking behaviour relates to the number of social associates. This would allow ecologists to determine whether there are social ‘personality’ types (Krause, James & Croft 2010; Wilson *et al*. 2012), and whether they are associated with a suite of non‐social behavioural traits, i.e. as part of a ‘behavioural syndrome’ (Sih *et al*. 2004; Sih, Chang & Wey 2014) or associated with ‘social carry‐over effects’ (Niemelä & Santostefano 2015). Furthermore, it has been observed that animal social groups show assortativity, where individuals of similar behavioural types are more likely to associate (Aplin *et al*. 2013; Wilson *et al*. 2014; Carter *et al*. 2015). This could result from ‘selection’ where individuals choose associates of a similar behavioural type, or ‘influence’ where individuals change their behavioural type to match that of their associates (Steglich, Snijders & West 2006; Burk, Steglich & Snijders 2007; Steglich, Snijders & Pearson 2010). Identifying exactly which processes are more influential will indicate the cognitive process occurring and therefore the selection pressures at work, yet cannot happen unless the change in traits is ordered in a dynamic analysis. Furthermore, assortativity can arise as a byproduct of triadic closure (Ilany & Akcay 2016), suggesting that proper control for this effect is necessary. Finally, the social niche hypothesis suggests that repeated social interactions lead to an increase in within‐individual consistency (Bergmüller & Taborsky 2010; Montiglio, Ferrari & Reale 2013). Evidence is mixed however (Laskowski & Bell 2014; Laskowski & Pruitt 2014; Modlmeier *et al*. 2014). Investigating this question further with SAOMs will allow broader trends to be identified.

## Future work

It is clear that SAOMs have great potential to inform the study of animal networks, but have potentially problematic assumptions and significant limitations. For many of these potential drawbacks, there is currently a lack of precise understanding of the impact they might have. Therefore, a fundamental next step is to test the susceptibility of SAOMs to type I and II errors in networks with a range of structures and constructed with a range of different interaction definitions, for a variety of missing levels of information. This work could use simulation‐modelling to determine the ability of SAOMs to detect a signal in simulated network data sets with different degrees of missing data (e.g. Huisman & Steglich 2008; Hipp *et al*. 2015).

It is also apparent that some of the current problems for using SAOMs with animal networks could be addressed by continued development of the modelling framework, or by making use of major advantages of SAOMs (e.g. the individual‐based approach) to develop or adapt alternative approaches specifically designed for animal networks. Many of the statistical processes used within a SAOM, such as Markov chains, are becoming increasingly familiar to ecologists and there is no reason why extensions, such as the ability to incorporate edge weights into the models, could not be developed specifically for animal networks. We hope that by highlighting this methodological tool we can stimulate further developments to enhance its utility.

## Summary

SAOMs are a potentially useful tool for studying animal social networks, and their use in ecology is increasing rapidly. By providing a review of their uses, and a practical guide in the Supporting Information, we hope to aid those interested in applying it to their own data. Appreciating the range of effects that can and have been implemented in SAOMs in other fields should enable ecologists to ask new questions of existing data sets or formulate new questions surrounding social and non‐social behaviour. However, there are still a number of key challenges that must be addressed. First, how well the key assumptions of SAOMs are satisfied in different animal study systems, and second, how SAOMs can be modified to improve their applicability to the types of data sets generated in ecological research. Satisfying these concerns, while exploring a range of network‐based questions in ecology promises to provide new insights into the relationships between social systems and broader evolutionary and ecological processes.

## Authors’ contributions

D.N.F. and M.J.S. conceived the idea for the manuscript, with D.N.F. leading the writing. All authors contributed to the writing of successive drafts. D.N.F. and T.T. contributed to collecting the data for the Supporting Information as members of the WildCrickets research group. D.N.F. analysed these data with contributions from A.I. and M.J.S. All authors gave final approval for publication.

## Data accessibility

This article used no data. For the data used as an example in the practical guide, see the online Supporting Information.

## Supporting information


**Figure S1.** SAOMs Practical guide. Flow chart.

**Figure S2–S4.** SAOMs Practical guide. RSiena bad GOF plots.

**Figure S5–S7.** SAOMs Practical guide. RSiena good GOF plots.

**Figure S8.** SAOMs Practical guide. Cricket social network.

**Data S1.** SAOMs Practical guide text.

**Data S2.** SAOMs R Code.

**Data S3.** SAOMs behaviour data.

**Data S4.** SAOMs network data 1.

**Data S5.** SAOMs network data 2.

**Data S6.** SAOMs network data 3.

**Data S7.** SAOMs sex data.

## References

[jane12630-bib-0001] Adelman, J.S. , Moyers, S.C. , Farine, D.R. & Hawley, D.M. (2015) Feeder use predicts both acquisition and transmission of a contagious pathogen in a North American songbird. Proceedings of the Royal Society B: Biological Sciences, 282, 20151429. 10.1098/rspb.2015.1429 PMC4614752 26378215

[jane12630-bib-0002] Allen, J. , Weinrich, M. , Hoppitt, W. & Rendell, L. (2013) Network‐based diffusion analysis reveals cultural transmission of lobtail feeding in humpback whales. Science, 340, 485–488. 23620054 10.1126/science.1231976

[jane12630-bib-0003] Apicella, C.L. , Marlowe, F.W. , Fowler, J.H. & Christakis, N.A. (2012) Social networks and cooperation in hunter‐gatherers. Nature, 481, 497–501. 22281599 10.1038/nature10736 PMC3340565

[jane12630-bib-0004] Aplin, L.M. , Farine, D.R. , Morand‐Ferron, J. & Sheldon, B.C. (2012) Social networks predict patch discovery in a wild population of songbirds. Proceedings of the Royal Society B: Biological Sciences, 279, 4199–4205. 10.1098/rspb.2012.1591 PMC3441092 22915668

[jane12630-bib-0005] Aplin, L.M. , Farine, D.R. , Morand‐Ferron, J. , Cole, E.F. , Cockburn, A. & Sheldon, B.C. (2013) Individual personalities predict social behaviour in wild networks of great tits (*Parus major*). Ecology Letters, 16, 1365–1372. 24047530 10.1111/ele.12181

[jane12630-bib-0006] Aplin, L.M. , Farine, D.R. , Morand‐Ferron, J. , Cockburn, A. , Thornton, A. & Sheldon, B.C. (2015a) Experimentally induced innovations lead to persistent culture via conformity in wild birds. Nature, 518, 538–541. 25470065 10.1038/nature13998 PMC4344839

[jane12630-bib-0007] Aplin, L.M. , Firth, J.A. , Farine, D.R. *et al* (2015b) Consistent individual differences in the social phenotypes of wild great tits, *Parus major* . Animal Behaviour, 108, 117–127. 26512142 10.1016/j.anbehav.2015.07.016 PMC4579410

[jane12630-bib-0008] Atton, N. , Hoppitt, W. , Webster, M.M. , Galef, B.G. & Laland, K.N. (2012) Information flow through threespine stickleback networks without social transmission. Proceedings of the Royal Society B: Biological Sciences, 279, 4272–4278. 10.1098/rspb.2012.1462 PMC3441084 22896644

[jane12630-bib-0009] Bascompte, J. (2007) Networks in ecology. Basic and Applied Ecology, 8, 485–490.

[jane12630-bib-0010] Bell, A.M. , Hankison, S.J. & Laskowski, K.L. (2009) The repeatability of behaviour: a meta‐analysis. Animal Behaviour, 77, 771–783. 24707058 10.1016/j.anbehav.2008.12.022 PMC3972767

[jane12630-bib-0011] Bergmüller, R. & Taborsky, M. (2010) Animal personality due to social niche specialisation. Trends in Ecology & Evolution, 25, 504–511. 20638151 10.1016/j.tree.2010.06.012

[jane12630-bib-0012] Best, E.C. , Dwyer, R.G. , Seddon, J.M. & Goldizen, A.W. (2014) Associations are more strongly correlated with space use than kinship in female eastern grey kangaroos. Animal Behaviour, 89, 1–10.

[jane12630-bib-0013] Blonder, B. & Dornhaus, A. (2011) Time‐ordered networks reveal limitations to information flow in ant colonies. PLoS ONE, 6, e20298. 21625450 10.1371/journal.pone.0020298 PMC3098866

[jane12630-bib-0014] Blonder, B. , Wey, T.W. , Dornhaus, A. , James, R. & Sih, A. (2012) Temporal dynamics and network analysis. Methods in Ecology and Evolution, 3, 958–972.

[jane12630-bib-0015] Blumstein, D.T. , Petelle, M.B. & Wey, T.W. (2013) Defensive and social aggression: repeatable but independent. Behavioral Ecology, 24, 457–461.

[jane12630-bib-0016] Boogert, N.J. , Reader, S.M. , Hoppitt, W. & Laland, K.N. (2008) The origin and spread of innovations in starlings. Animal Behaviour, 75, 1509–1518.

[jane12630-bib-0017] Boogert, N.J. , Nightingale, G.F. , Hoppitt, W. & Laland, K.N. (2014) Perching but not foraging networks predict the spread of novel foraging skills in starlings. Behavioural Processes, 109, 135–144. 25178191 10.1016/j.beproc.2014.08.016

[jane12630-bib-0018] Borgeaud, C. , Sosa, S. , Sueur, C. , Bshary, R. & van de Waal, E. (2016) Intergroup variation of social relationships in wild vervet monkeys: a dynamic network approach. Frontiers in Psychology, 7, 915. 27445890 10.3389/fpsyg.2016.00915 PMC4914564

[jane12630-bib-0019] Boucherie, P.H. , Sosa, S. , Pasquaretta, C. & Dufour, V. (2016) A longitudinal network analysis of social dynamics in rooks *Corvus frugilegus*: repeated group modifications do not affect social network in captive rooks. Current Zoology, zow083. 10.1093/cz/zow083 PMC5804189 29491998

[jane12630-bib-0020] Brent, L.J.N. , Heilbronner, S.R. , Horvath, J.E. , Gonzalez‐Martinez, J. , Ruiz‐Lambides, A. , Robinson, A.G. , Skene, J.H.P. & Platt, M.L. (2013) Genetic origins of social networks in rhesus macaques. Scientific Reports, 3, 1042. 23304433 10.1038/srep01042 PMC3540398

[jane12630-bib-0021] Bull, C.M. , Godfrey, S.S. & Gordon, D.M. (2012) Social networks and the spread of *Salmonella* in a sleepy lizard population. Molecular Ecology, 21, 4386–4392. 22845647 10.1111/j.1365-294X.2012.05653.x

[jane12630-bib-0022] Burk, W.J. , Steglich, C.E.G. & Snijders, T.A.B. (2007) Beyond dyadic interdependence: actor‐oriented models for co‐evolving social networks and individual behaviors. International Journal of Behavioral Development, 31, 397–404.

[jane12630-bib-0023] Carter, K.D. , Seddon, J.M. , Frère, C.H. , Carter, J.K. & Goldizen, A.W. (2013) Fission–fusion dynamics in wild giraffes may be driven by kinship, spatial overlap and individual social preferences. Animal Behaviour, 85, 385–394.

[jane12630-bib-0024] Carter, A.J. , Lee, A.E.G. , Marshall, H.H. , Tico, M.T. & Cowlishaw, G. (2015) Phenotypic assortment in wild primate networks: implications for the dissemination of information. Royal Society Open Science, 2, 140444. 26064652 10.1098/rsos.140444 PMC4453262

[jane12630-bib-0025] Croft, D.P. , Darden, S.K. & Wey, T.W. (2016) Current directions in animal social networks. Current Opinion in Behavioral Sciences, 12, 52–58.

[jane12630-bib-0026] Croft, D.P. , James, R. & Krause, J. (2008) Exploring Animal Social Networks. Princeton University Press, Oxford, UK.

[jane12630-bib-0027] Croft, D.P. , Madden, J.R. , Franks, D.W. & James, R. (2011) Hypothesis testing in animal social networks. Trends in Ecology & Evolution, 26, 502–507. 21715042 10.1016/j.tree.2011.05.012

[jane12630-bib-0028] Dall, S.R.X. , Houston, A.I. & McNamara, J.M. (2004) The behavioural ecology of personality: consistent individual differences from an adaptive perspective. Ecology Letters, 7, 734–739.

[jane12630-bib-0029] Dey, C.J. , Reddon, A.R. , O'Connor, C.M. & Balshine, S. (2013) Network structure is related to social conflict in a cooperatively breeding fish. Animal Behaviour, 85, 395–402.

[jane12630-bib-0030] Dey, C.J. , Tan, Q.Y.J. , O'Connor, C.M. , Reddon, A.R. , Caldwell, J.R. & Balshine, S. (2015) Dominance network structure across reproductive contexts in the cooperatively breeding cichlid fish *Neolamprologus pulcher* . Current Zoology, 61, 45–54.

[jane12630-bib-0031] Farine, D.R. (2014) Measuring phenotypic assortment in animal social networks: weighted associations are more robust than binary edges. Animal Behaviour, 89, 141–153.

[jane12630-bib-0035] Farine, D.R. , Aplin, L.M. , Sheldon, B.C. & Hoppitt, W. (2015) Interspecific social networks promote information transmission in wild songbirds. Proceedings of the Royal Society B: Biological Sciences, 282, 20142804. 10.1098/rspb.2014.2804 PMC4345451 25673683

[jane12630-bib-0032] Farine, D.R. , Montiglio, P.‐O. & Spiegel, O. (2015) From individuals to groups and back: the evolutionary implications of group phenotypic composition. Trends in Ecology & Evolution, 30, 609–621. 26411618 10.1016/j.tree.2015.07.005 PMC4594155

[jane12630-bib-0033] Farine, D.R. & Strandburg‐Peshkin, A. (2015) Estimating uncertainty and reliability of social network data using Bayesian inference. Royal Society Open Science, 2, 150367. 26473059 10.1098/rsos.150367 PMC4593693

[jane12630-bib-0034] Farine, D.R.D. & Whitehead, H. (2015) Constructing, conducting, and interpreting animal social network analysis. The Journal of Animal Ecology, 84, 1144–1163. 26172345 10.1111/1365-2656.12418 PMC4973823

[jane12630-bib-0036] Firth, J.A. & Sheldon, B.C. (2015) Experimental manipulation of avian social structure reveals segregation is carried over across contexts. Proceedings of the Royal Society B: Biological Sciences, 282, 20142350. 10.1098/rspb.2014.2350 PMC4344146 25652839

[jane12630-bib-0037] Firth, J. & Sheldon, B. (2016) Social carry‐over effects underpin trans‐seasonally linked structure in a wild bird population. Ecology Letters, 19, 1324–1332. 27623746 10.1111/ele.12669 PMC5082527

[jane12630-bib-0038] Fisher, D.N. , Rodríguez‐Muñoz, R. & Tregenza, T. (2016a) Comparing pre‐ and post‐copulatory mate competition using social network analysis in wild crickets. Behavioral Ecology, 27, 912–919. 27174599 10.1093/beheco/arv236 PMC4863196

[jane12630-bib-0039] Fisher, D.N. , Rodríguez‐Muñoz, R. & Tregenza, T. (2016b) Wild cricket social networks show stability across generations. BMC Evolutionary Biology, 16, 151. 27464504 10.1186/s12862-016-0726-9 PMC4964091

[jane12630-bib-0040] Formica, V.A. , McGlothlin, J.W. , Wood, C.W. , Augat, M.E. , Butterfield, R.E. , Barnard, M.E. & Brodie, E.D. (2011) Phenotypic assortment mediates the effect of social selection in a wild beetle population. Evolution, 65, 2771–2781. 21967420 10.1111/j.1558-5646.2011.01340.x

[jane12630-bib-0041] Formica, V. , Wood, C. , Cook, P. & Brodie, E. (2016) Consistency of animal social networks after disturbance. Behavioral Ecology, arw128.

[jane12630-bib-0042] Franks, D.W. , Ruxton, G.D. & James, R. (2009) Sampling animal association networks with the gambit of the group. Behavioral Ecology and Sociobiology, 64, 493–503.

[jane12630-bib-0043] Frère, C.H. , Krützen, M. , Mann, J. , Watson‐Capps, J.J. , Tsai, Y.J. , Patterson, E.M. , Connor, R. , Bejder, L. & Sherwin, W.B. (2010) Home range overlap, matrilineal and biparental kinship drive female associations in bottlenose dolphins. Animal Behaviour, 80, 481–486.

[jane12630-bib-0044] Granovetter, M. (1973) The strength of weak ties. American Journal of Sociology, 78, 1360–1380.

[jane12630-bib-0045] Greenan, C.C. (2015) Diffusion of innovations in dynamic networks. Journal of the Royal Statistical Society: Series A (Statistics in Society), 178, 147–166.

[jane12630-bib-0046] Hamede, R.K. , Bashford, J. , McCallum, H. & Jones, M. (2009) Contact networks in a wild *Tasmanian devil* (*Sarcophilus harrisii*) population: using social network analysis to reveal seasonal variability in social behaviour and its implications for transmission of devil facial tumour disease. Ecology Letters, 12, 1147–1157. 19694783 10.1111/j.1461-0248.2009.01370.x

[jane12630-bib-0047] Hipp, J.R. , Wang, C. , Butts, C.T. , Jose, R. & Lakon, C.M. (2015) Research Note: the consequences of different methods for handling missing network data in Stochastic Actor Based Models. Social Networks, 41, 56–71. 25745276 10.1016/j.socnet.2014.12.004 PMC4346092

[jane12630-bib-0048] Hobaiter, C. , Poisot, T. , Zuberbühler, K. , Hoppitt, W. & Gruber, T. (2014) Social network analysis shows direct evidence for social transmission of tool use in wild chimpanzees. PLoS Biology, 12, e1001960. 25268798 10.1371/journal.pbio.1001960 PMC4181963

[jane12630-bib-0049] Huisman, M. & Steglich, C. (2008) Treatment of non‐response in longitudinal network studies. Social Networks, 30, 297–308.

[jane12630-bib-0050] Ilany, A. & Akcay, E. (2016) Social inheritance can explain the structure of animal social networks. Nature Communications, 7, 12084. 10.1038/ncomms12084 PMC4931239 27352101

[jane12630-bib-0051] Ilany, A. , Booms, A.S. & Holekamp, K.E. (2015) Topological effects of network structure on long‐term social network dynamics in a wild mammal. Ecology Letters, 18, 687–695. 25975663 10.1111/ele.12447 PMC4486283

[jane12630-bib-0052] Jacoby, D.M.P. , Fear, L.N. , Sims, D.W. & Croft, D.P. (2014) Shark personalities? Repeatability of social network traits in a widely distributed predatory fish. Behavioral Ecology and Sociobiology, 68, 1995–2003.

[jane12630-bib-0053] Jeanson, R. (2012) Long‐term dynamics in proximity networks in ants. Animal Behaviour, 83, 915–923.

[jane12630-bib-0054] Jones, H.C. (2011) Social Network Analysis of Behavioural Interactions Influencing the Development of Fin Damage in Atlantic Salmon (Salmo salar). University of Cambridge, Cambridge, UK.

[jane12630-bib-0055] Koolhaas, J.M. , Korte, S.M. , De Boer, S.F. , Van Der Vegt, B.J. , Van Reenen, C.G. , Hopster, H. , De Jong, I.C. , Ruis, M.A. & Blokhuis, H.J. (1999) Coping styles in animals: current status in behavior and stress‐physiology. Neuroscience and Biobehavioral Reviews, 23, 925–935. 10580307 10.1016/s0149-7634(99)00026-3

[jane12630-bib-0056] Kossinets, G. (2006) Effects of missing data in social networks. Social Networks, 28, 247–268.

[jane12630-bib-0057] Kossinets, G. & Watts, D.J. (2006) Empirical analysis of an evolving social network. Science, 311, 88–90. 16400149 10.1126/science.1116869

[jane12630-bib-0058] Krause, J. , James, R. & Croft, D.P. (2010) Personality in the context of social networks. Philosophical Transactions of the Royal Society of London Series B, Biological Sciences, 365, 4099–4106. 21078661 10.1098/rstb.2010.0216 PMC2992749

[jane12630-bib-0059] Krivitsky, P. (2012) Exponential‐family random graph models for valued networks. Electronic Journal of Statistics, 6, 1100–1128. 24678374 10.1214/12-EJS696 PMC3964598

[jane12630-bib-0060] Kurvers, R.H.J.M. , Krause, J. , Croft, D.P. , Wilson, A.D.M. & Wolf, M. (2014) The evolutionary and ecological consequences of animal social networks: emerging issues. Trends in Ecology & Evolution, 29, 326–335. 24792356 10.1016/j.tree.2014.04.002

[jane12630-bib-0061] Laskowski, K.L. & Bell, A.M. (2014) Strong personalities, not social niches, drive individual differences in social behaviours in sticklebacks. Animal Behaviour, 90, 287–295. 25076789 10.1016/j.anbehav.2014.02.010 PMC4112482

[jane12630-bib-0062] Laskowski, K.L. & Pruitt, J.N. (2014) Evidence of social niche construction: persistent and repeated social interactions generate stronger personalities in a social spider. Proceedings of the Royal Society B: Biological Sciences, 281, 20133166. 10.1098/rspb.2013.3166 PMC3996602 24671972

[jane12630-bib-0063] Lehmann, H. (2009) On the Applicability of Agent Based Modelling in Behavioural Ecology. University of Bath, Bath, UK.

[jane12630-bib-0064] Lusseau, D. , Whitehead, H. & Gero, S. (2008) Incorporating uncertainty into the study of animal social networks. Animal Behaviour, 75, 1809–1815.

[jane12630-bib-0065] McDonald, J.L. , Smith, G.C. , McDonald, R.A. , Delahay, R.J. & Hodgson, D. (2014) Mortality trajectory analysis reveals the drivers of sex‐specific epidemiology in natural wildlife‐disease interactions. Proceedings of the Royal Society B: Biological Sciences, 281, 20140526. 10.1098/rspb.2014.0526 PMC4123697 25056621

[jane12630-bib-0066] Modlmeier, A.P. , Laskowski, K.L. , DeMarco, A.E. , Coleman, A. , Zhao, K. , Brittingham, H.A. , McDermott, D.R. & Pruitt, J.N. (2014) Persistent social interactions beget more pronounced personalities in a desert‐dwelling social spider. Biology Letters, 10, 20140419. 25165452 10.1098/rsbl.2014.0419 PMC4155910

[jane12630-bib-0067] Montiglio, P.‐O. , Ferrari, C. & Reale, D. (2013) Social niche specialization under constraints: personality, social interactions and environmental heterogeneity. Philosophical Transactions of the Royal Society B: Biological Sciences, 368, 20120343. 10.1098/rstb.2012.0343 PMC3638446 23569291

[jane12630-bib-0068] Niemelä, P.T. & Santostefano, F. (2015) Social carry‐over effects on non‐social behavioral variation: mechanisms and consequences. Frontiers in Ecology and Evolution, 3, 49.

[jane12630-bib-0069] Oh, K.P. & Badyaev, A.V. (2010) Structure of social networks in a passerine bird: consequences for sexual selection and the evolution of mating strategies. The American Naturalist, 176, E80–E89. 10.1086/655216 20608873

[jane12630-bib-0070] Pasquaretta, C. , Klenschi, E. , Pansanel, J. , Battesti, M. , Mery, F. & Sueur, C. (2016) Understanding dynamics of information transmission in *Drosophila melanogaster* using a statistical modeling framework for longitudinal network data (the RSiena package). Frontiers in Psychology, 7, 1–11. 27148146 10.3389/fpsyg.2016.00539 PMC4835720

[jane12630-bib-0071] Pinter‐Wollman, N. , Hobson, E. , Smith, J. *et al* (2013) The dynamics of animal social networks: analytical, conceptual, and theoretical advances. Behavioral Ecology, 25, 242–255.

[jane12630-bib-0072] Proulx, S.R. , Promislow, D.E.L. & Phillips, P.C. (2005) Network thinking in ecology and evolution. Trends in Ecology & Evolution, 20, 345–353. 16701391 10.1016/j.tree.2005.04.004

[jane12630-bib-0073] Ramos‐Fernández, G. , Boyer, D. & Gómez, V.P. (2006) A complex social structure with fission–fusion properties can emerge from a simple foraging model. Behavioral Ecology and Sociobiology, 60, 536–549.

[jane12630-bib-0074] Réale, D. , Reader, S.M. , Sol, D. , McDougall, P.T. & Dingemanse, N.J. (2007) Integrating animal temperament within ecology and evolution. Biological Reviews, 82, 291–318. 17437562 10.1111/j.1469-185X.2007.00010.x

[jane12630-bib-0075] Ripley, R.M. , Snijders, T.A.B. , Boda, Z. , Voros, A. & Preciado, P. (2015) Manual for SIENA Version 4.0 (Version October 10, 2015). University of Oxford, Oxford, UK.

[jane12630-bib-0076] Shizuka, D. , Chaine, A.S. , Anderson, J. , Johnson, O. , Laursen, I.M. & Lyon, B.E. (2014) Across‐year social stability shapes network structure in wintering migrant sparrows. Ecology Letters, 17, 998–1007. 24894316 10.1111/ele.12304

[jane12630-bib-0077] Shorrocks, B. & Croft, D. (2009) Necks and networks: a preliminary study of population structure in the reticulated giraffe (*Giraffa camelopardalis* reticulata de Winston). African Journal of Ecology, 47, 374–381.

[jane12630-bib-0078] Sih, A. , Chang, A.T. & Wey, T.W. (2014) Effects of behavioural type, social skill and the social environment on male mating success in water striders. Animal Behaviour, 94, 9–17.

[jane12630-bib-0079] Sih, A. , Bell, A.M. , Johnson, J.C. & Ziemba, R.E. (2004) Behavioral syndromes: an intergrative overiew. The Quarterly Review of Biology, 79, 241–277. 15529965 10.1086/422893

[jane12630-bib-0080] Silk, M.J. , Jackson, A.L. , Croft, D.P. , Colhoun, K. & Bearhop, S. (2015) The consequences of unidentifiable individuals for the analysis of an animal social network. Animal Behaviour, 104, 1–11.

[jane12630-bib-0081] Smith, B.R. & Blumstein, D.T. (2008) Fitness consequences of personality: a meta‐analysis. Behavioral Ecology, 19, 448–455.

[jane12630-bib-0082] Smith, J.A. & Moody, J. (2013) Structural effects of network sampling coverage I: nodes missing at random. Social Networks, 35, 652–668. 10.1016/j.socnet.2013.09.003 PMC3846431 24311893

[jane12630-bib-0083] Smith, J.A. , Moody, J. & Morgan, J.H. (2017) Network sampling coverage II: the effect of non‐random missing data on network measurement. Social Networks, 48, 78–99. 27867254 10.1016/j.socnet.2016.04.005 PMC5110009

[jane12630-bib-0084] Snijders, T.A.B. (2011) Statistical models for social networks. Annual Review of Sociology, 37, 131–153.

[jane12630-bib-0085] Snijders, T.A.B. , van de Bunt, G.G. & Steglich, C.E.G. (2010) Introduction to stochastic actor‐based models for network dynamics. Social Networks, 32, 44–60.

[jane12630-bib-0086] Spiegel, O. , Leu, S.T. , Sih, A. & Bull, C.M. (2016) Socially interacting or indifferent neighbours? Randomization of movement paths to tease apart social preference and spatial constraints (ed T Münkemüller). Methods in Ecology and Evolution, 7, 971–979.

[jane12630-bib-0087] Steglich, C. , Snijders, T.A.B. & Pearson, M. (2010) Dynamic networks and behaviour: separating selection from influence. Sociological Methodology, 40, 329–393.

[jane12630-bib-0088] Steglich, C. , Snijders, T. & West, P. (2006) Applying SIENA: an illustrative analysis of the coevolution of adolescents’ friendship networks, taste in music, and alcohol consumption. Methodology: European Journal of Research Methods for the Behavioral and Social Sciences, 2, 48–56.

[jane12630-bib-0089] Sundaresan, S.R. , Fischhoff, I.R. , Dushoff, J. & Rubenstein, D.I. (2007) Network metrics reveal differences in social organization between two fission‐fusion species, Grevy's zebra and onager. Oecologia, 151, 140–149. 16964497 10.1007/s00442-006-0553-6

[jane12630-bib-0090] Tompkins, D.M. , Dunn, A.M. , Smith, M.J. & Telfer, S. (2011) Wildlife diseases: from individuals to ecosystems. The Journal of Animal Ecology, 80, 19–38. 20735792 10.1111/j.1365-2656.2010.01742.x

[jane12630-bib-0091] Van der Waal, K.L. , Atwill, E.R. , Isbell, L.A. & McCowan, B. (2014) Linking social and pathogen transmission networks using microbial genetics in giraffe (*Giraffa camelopardalis*). The Journal of Animal Ecology, 83, 406–414. 24117416 10.1111/1365-2656.12137

[jane12630-bib-0092] Voelkl, B. & Kasper, C. (2009) Social structure of primate interaction networks facilitates the emergence of cooperation. Biology Letters, 5, 462–464. 19443505 10.1098/rsbl.2009.0204 PMC2781925

[jane12630-bib-0093] Wang, C. , Butts, C.T. , Hipp, J.R. , Jose, R. & Lakon, C.M. (2016) Multiple imputation for missing edge data: a predictive evaluation method with application to add health. Social Networks, 45, 89–98. 26858508 10.1016/j.socnet.2015.12.003 PMC4743534

[jane12630-bib-0094] Weber, N. , Carter, S.P. , Dall, S.R.X. , Delahay, R.J. , McDonald, J.L. , Bearhop, S. & McDonald, R.A. (2013) Badger social networks correlate with tuberculosis infection. Current Biology, 23, R915–R916. 24156807 10.1016/j.cub.2013.09.011

[jane12630-bib-0095] Whitehead, H. (2008) Analysing Animal Societies: Quantitative Methods for Vertebrate Social Analysis. The University Chicago Press, Chicago, IL, USA.

[jane12630-bib-0096] Whitehead, H. & Dufault, S. (1999) Techniques for analyzing vertebrate social structure using identified individuals: review and recommendations Advances in the Study of Behavior, 28th edn (eds Slater P., Rosenblatt J. & Roper T.), pp. 33–73. Academic Press, San Diego, CA, USA.

[jane12630-bib-0097] Whitehead, H. & James, R. (2015) Generalized affiliation indices extract affiliations from social network data. Methods in Ecology and Evolution, 6, 836–844.

[jane12630-bib-0098] Wilson, A.D.M. , Krause, S. , Dingemanse, N.J. & Krause, J. (2012) Network position: a key component in the characterization of social personality types. Behavioral Ecology and Sociobiology, 67, 163–173.

[jane12630-bib-0099] Wilson, A.D.M. , Krause, S. , James, R. , Croft, D.P. , Ramnarine, I.W. , Borner, K.K. , Clement, R.J.G. & Krause, J. (2014) Dynamic social networks in guppies (*Poecilia reticulata*). Behavioral Ecology and Sociobiology, 68, 915–925.

[jane12630-bib-0100] Wolf, M. & Weissing, F.J. (2012) Animal personalities: consequences for ecology and evolution. Trends in Ecology & Evolution, 27, 452–461. 22727728 10.1016/j.tree.2012.05.001

[jane12630-bib-0101] Wölfer, R. , Faber, N.S. & Hewstone, M. (2015) Social network analysis in the science of groups: cross‐sectional and longitudinal applications for studying intra‐ and intergroup behavior. Group Dynamics: Theory, Research, and Practice, 19, 45–61.

[jane12630-bib-0102] Zuber, F. (2014) Spread of unethical behavior in organizations: a dynamic social network perspective. Journal of Business Ethics, 131, 151–172.

