# Supplementary figures and images for: Analysing animal social network dynamics: the potential of stochastic actor‐oriented models
Source: J Anim Ecol. 2017 Feb 1;86(2):202–12. doi: 10.1111/1365-2656.12630 (PMC6849756; doi:10.1111/1365-2656.12630)

# Start

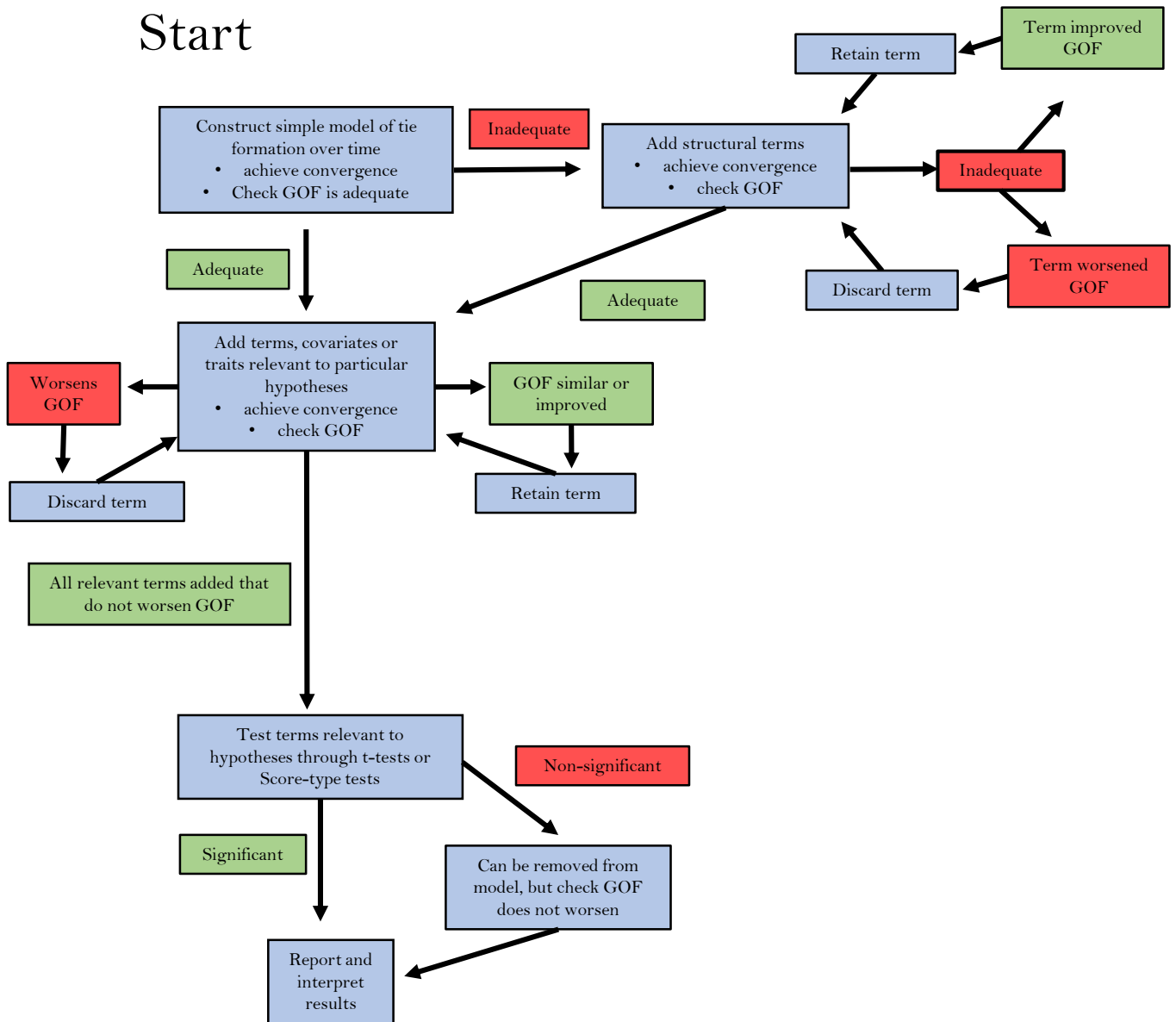

# End

Supplement: Supplementary file 1 — Figure S1. SAOMs Practical guide. Flow chart. [file JANE-86-202-s001.pdf]

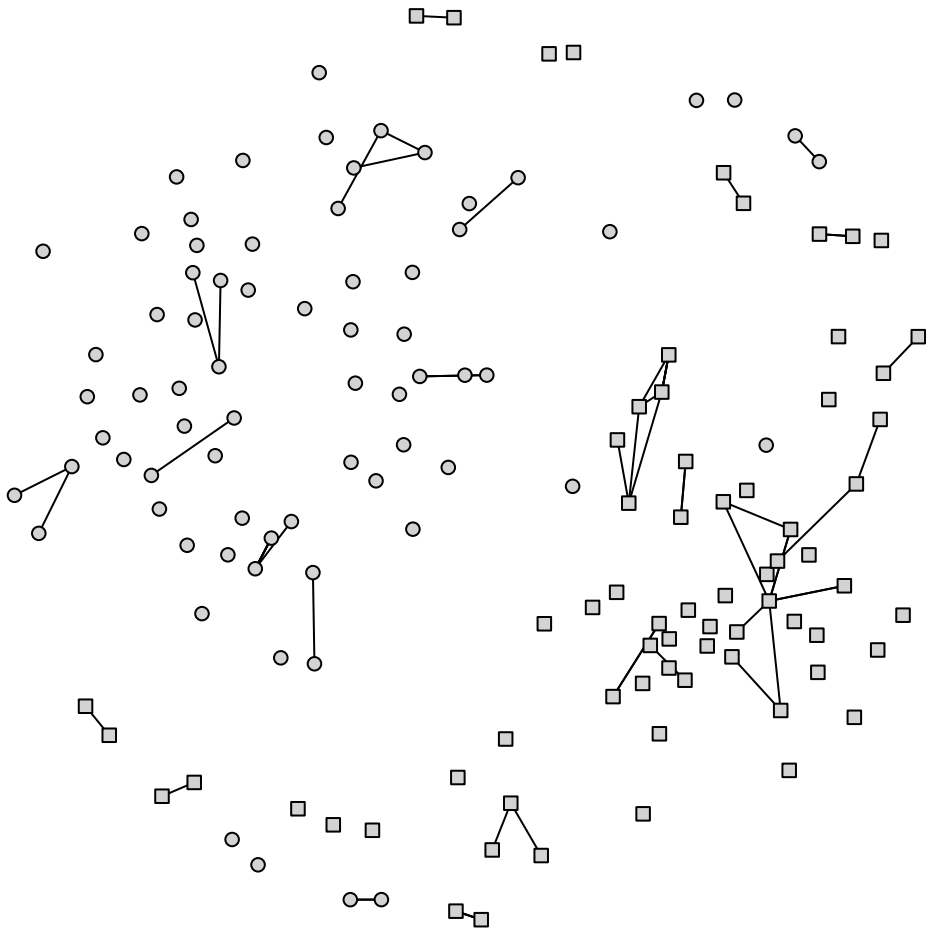

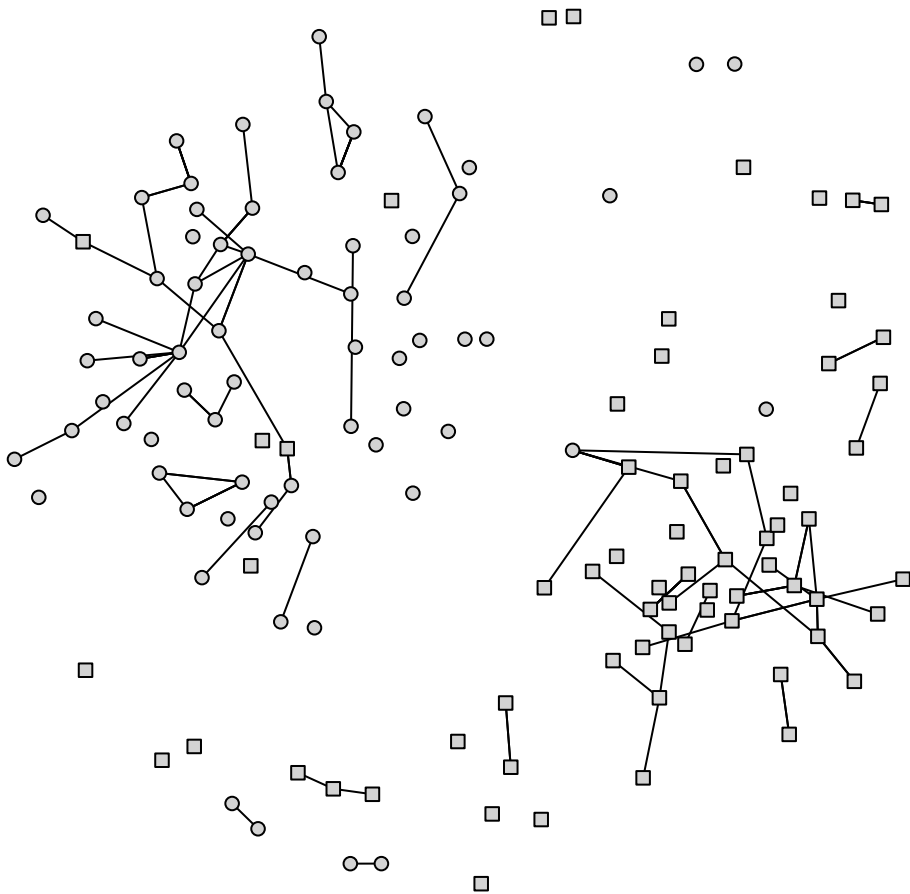

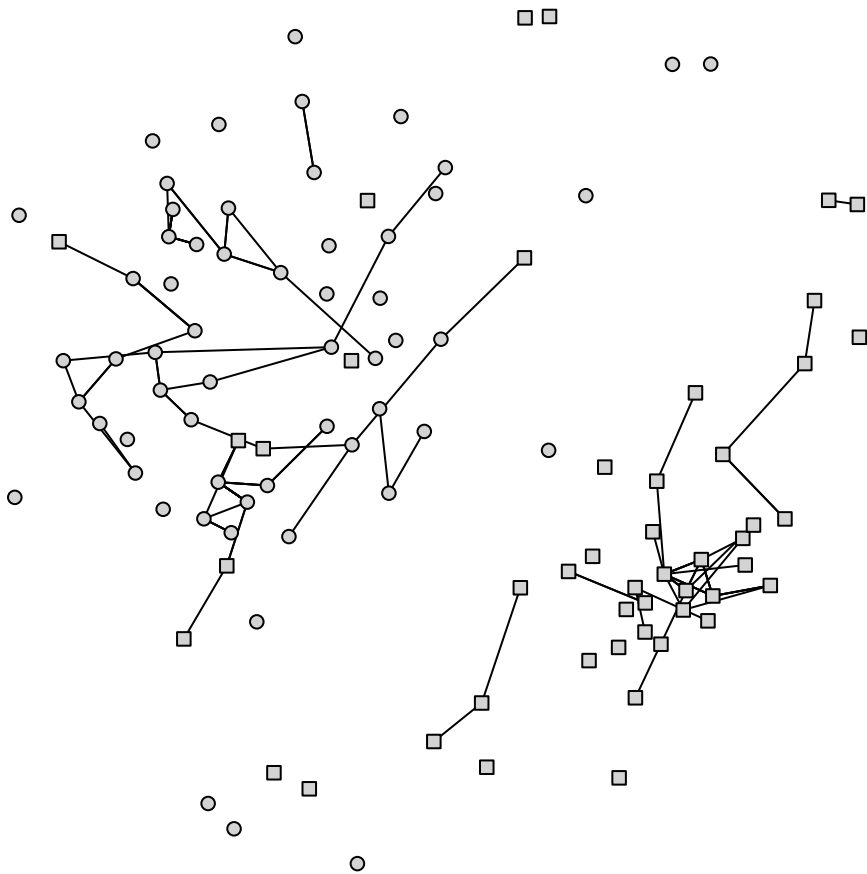

Supplement: Supplementary file 4 — Figure S8. SAOMs Practical guide. Cricket social network. [file JANE-86-202-s004.pdf]
