# Supplementary material for: Analysing animal social network dynamics: the potential of stochastic actor‐oriented models
Source: J Anim Ecol. 2017 Feb 1;86(2):202–12. doi: 10.1111/1365-2656.12630 (PMC6849756; doi:10.1111/1365-2656.12630)
Supplement: Supplementary file 2 — Figure S2–S4. SAOMs Practical guide. RSiena bad GOF plots. [file JANE-86-202-s002.pdf]

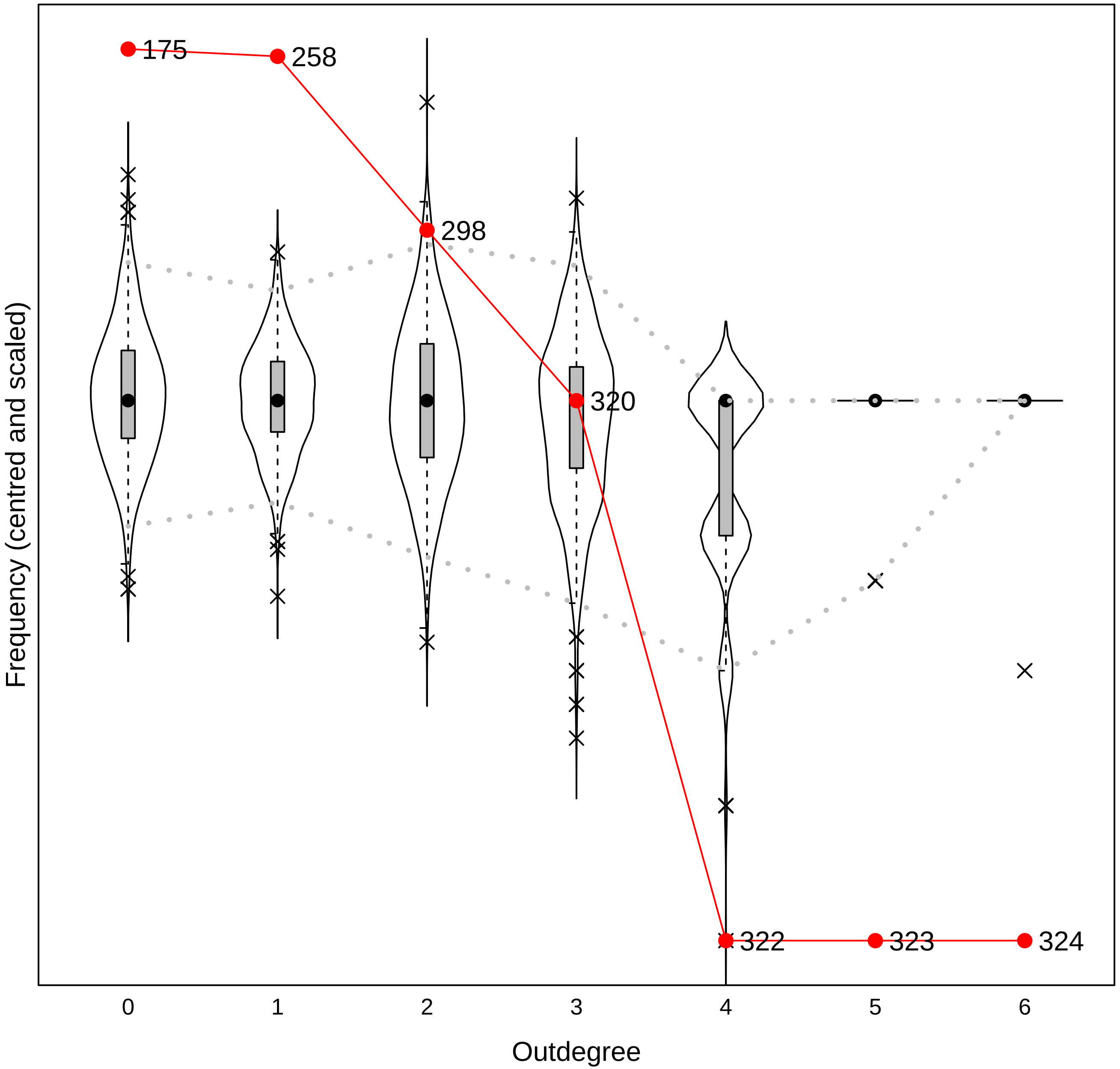

175
258
298
320
322
323
324
Frequency (centred and scaled)
0
1
2
3
4
5
6
Outdegree

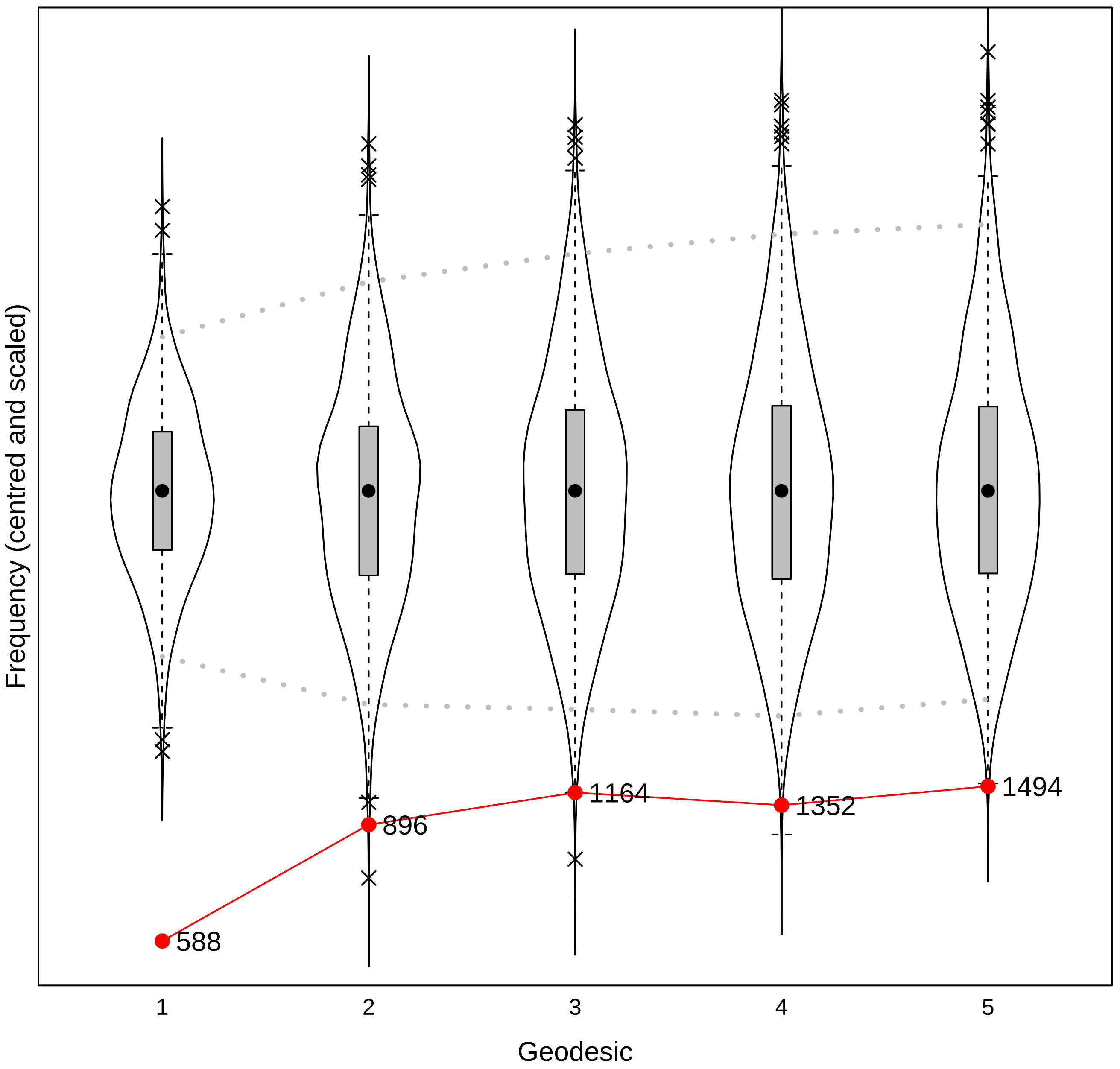

Frequency (centred and scaled)
Geodesic
1
2
3
4
5
588
896
1164
1352
1494

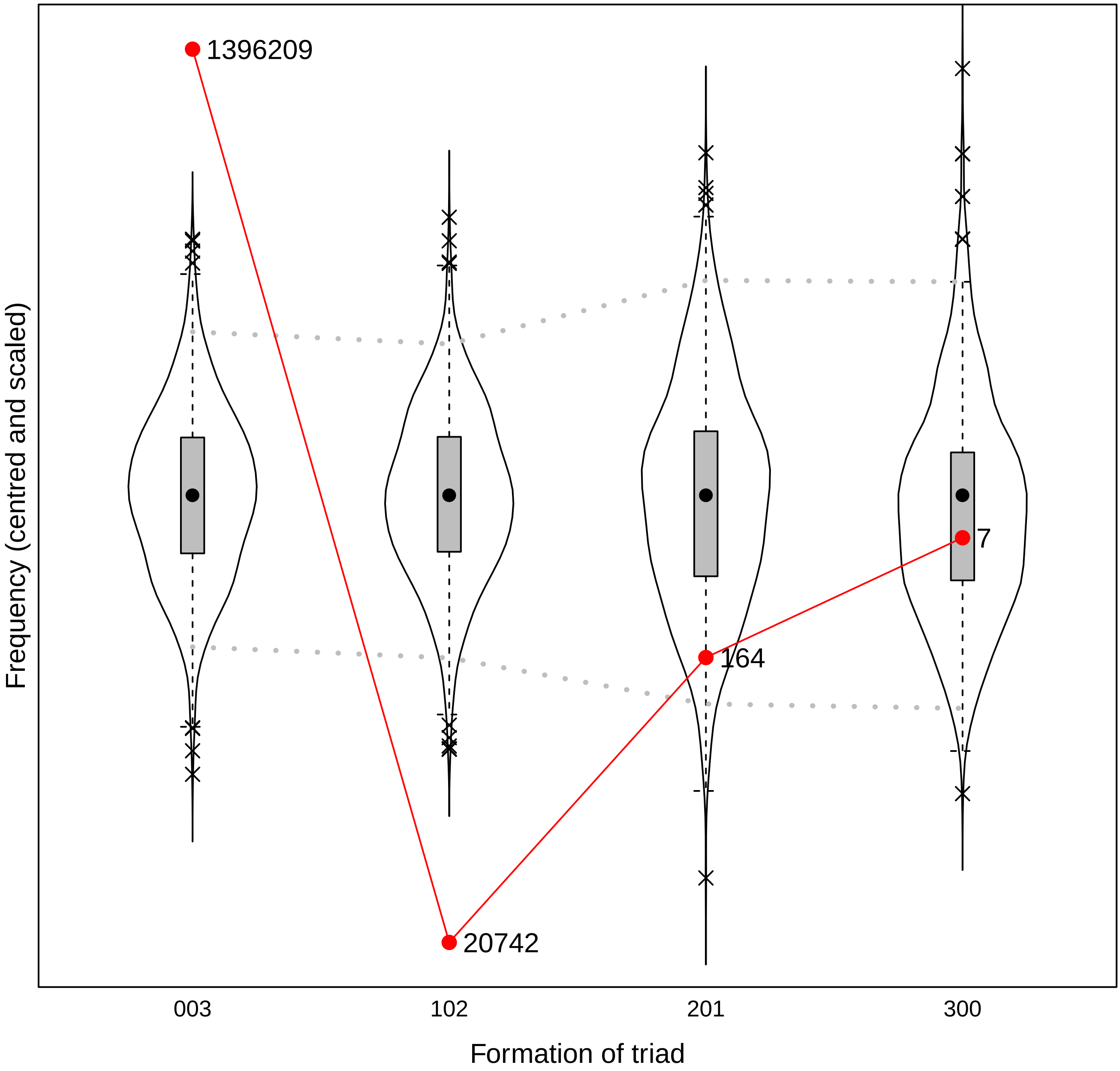

1396209
20742
164
7
Frequency (centred and scaled)
003
102
201
300
Formation of triad
