# Supplementary material for: Analysing animal social network dynamics: the potential of stochastic actor‐oriented models
Source: J Anim Ecol. 2017 Feb 1;86(2):202–12. doi: 10.1111/1365-2656.12630 (PMC6849756; doi:10.1111/1365-2656.12630)
Supplement: Supplementary file 5 — Data S1. SAOMs Practical guide text. [file JANE-86-202-s005.docx]

Analysing animal social network dynamics: A practical guide to stochastic actor-orientated models

## Overview

This document sits alongside our review on the application of stochastic actor-orientated models (SAOMs) for ecologists. In it we provide a guide as to the data requirements, model fitting and inference for a SAOM. We then provide an example using a dataset of the social interactions of field crickets (*Gryllus campestris*) complete with an annotated R script. This will allow those interested to re-create the analysis we describe, and may function as a useful starting point for those wishing to conduct their own analyses.

## Data types

For examples of some of these types of data see the data supplied alongside this guide.

The starting point is the network data:

- *t* matrices of size *n* x *n*, representing all members of the population (*n*) and their observed social associations at each time point (*t*).

Ties can be directed or undirected; this will influence what network processes can be investigated. Individuals do not need to be able to interact at every time point to be included. If they could not interact at a particular time point (e.g. they had died or left the study area) “structural zeroes” can be entered into the rows and columns for that individual in the association matrix at that time point (Ripley *et al.* 2015). This prevents the lack of interactions involving that individual informing parameter estimates and avoids the omission of individuals that were not socially active at every time point (e.g. Frère et al 2010; Aplin et al 2014). Excessive structural zeroes which would reduce the effective group size below the guideline size below should be avoided however as they can reduce the stability of parameter estimates. “Structural ones” can also be entered, indicating individuals that must interact (e.g. mothers and their offspring), and therefore that these interactions should not influence parameter estimates. As discussed in the main text, associations between individuals are represented as either existing or not i.e. they are binary, although a small set of different interaction strengths can be entered through the use of multiple ordered networks.

Alongside the network data, various predictor variables can be added:

- *n* constant actor covariates (e.g. sex)
- *n* x *n* matrices of non-changing dyadic covariates (e.g. genetic relatedness)
- *t*-1 changing environmental conditions (e.g. rainfall)
- *t*-1 *n* changing actor covariates (e.g. body condition)
- *t*-1 *n* x *n* matrices of changing dyadic covariates (e.g. spatial proximity)

These covariates can be categorical or continuous. There are *t*-1 values for the changing covariates as SAOMs model the change between *t* and *t*+1. The effect of parameters on the structure of the network at *t* = 1 is not estimated and so covariate values are not required.

Finally, one can model changing traits as response variables:

- matrices of *n* x *t* trait values.

These must be categorical or ordinal data, with a recommended 2-5 categories (Ripley *et al.* 2015). This requirement is not overly restrictive: infection status, whether an individual has acquired a piece of information or not, body condition indices, or the level of some behaviour e.g. degree of risk-taking can all be accommodated within this limitation.

## Data requirements

A SAOM with RSiena provides no means to account directly for data uncertainty. Therefore, accurate data for each of the variables is required. This means that in situations where there is a medium-high edge uncertainty, or where traits cannot be measured accurately, e.g. for some disease diagnoses, a SAOM will not be appropriate. However, given the advances in modern technology allowing (often automated) high-resolution data collection, the data requirements for a SAOM through RSiena will increasingly be met by animal network or animal behaviour datasets. The following serves as guidelines, for further details see Snijders *et al*. (2010):

- At least two networks (interactions or associations over different time periods) are required. Three are required if a behavioural change is also modelled alongside a change in network structure (Ripley *et al.* 2015). Modelling many observations (more than 10) relies on the assumption that each effect is constant over time, or an interaction between the effect and time is included in the model.
- The number of individuals should be more than 20, although if a large number of observations are made then fewer may be acceptable. Networks with a large (>100) number of individuals require that each individual could theoretically interact with any other individual in the network. This may restrict the application of SAOMs in some animal systems, but controlling for this to some extent is possible with structural zeroes, or by including group membership or spatial information as covariates (see below).
- There is a minimum amount of change required in the network for change to be effectively modelled. Over all observations, a total of 40 changes (ties formed or dissolved) serves as a minimum. However, too high a number of changes per individual would violate the assumption that it is a gradually changing network. Whether too much change has occurred can be evaluated with the Jaccard index (Jaccard 1901). This is the ratio between the total number of ties present in both observations and the sum of: 1) the number of ties present in both observations; 2) the number of ties broken and 3) the number of ties created. A Jaccard index greater than 0.3 is desirable; models can be specified with lower indices but stable estimation may be hard to achieve.
- As mentioned above, missing data in the form of individuals entering and leaving the network over time can be handled by SAOMs. Missing data due to non-observation in the social network, other traits and covariates is also acceptable (coded as “NA” in R), providing that missingness is unbiased. The missing data is imputed typically using the last recorded value to allow the simulations to run, but then the imputed values do not inform parameter estimates (Ripley *et al.* 2015). Too great an amount of missing data (>20% missing, with <10% preferable) is however undesirable as it makes estimation less stable (Huisman & Steglich 2008; Ripley *et al.* 2015; see Wang 2007; Hipp *et al.* 2015; Wang *et al.* 2016 for further discussion).

## The modelling process

The total range of possible model specifications is daunting. Furthermore, the most complex model may be a poor starting point if it fails to converge or fits the data poorly, giving unreliable parameter estimates and therefore making the testing of any specific terms unwise. Hence the typical (and recommended) approach is to start with a basic model, such as the tendency for individuals to form ties with those with whom they share a mutual connection (triadic closure), then add more complex effects (Burk, Steglich & Snijders 2007; Snijders *et al.* 2010). Regardless of your modelling philosophy, terms with weak/non-significant effects may have to be removed from models, as large standard errors can lead to poor convergence. See the RSiena manual (Ripley *et al.* 2015), the SIENA website (<http://www.stats.ox.ac.uk/~snijders/siena/>) or our accompanying R code for how to specify effects and test them in R. Additionally, for undirected networks, the model type must be set. This states how two individuals become connected. For example, a tie may require agreement within the pair (e.g. in non-coercive mating). In contrast, in some cases a tie would be defined using a forcing model, where one individual forces the connection with the other (e.g. where a fight is motivated by only one contestant). For further information on model types see the RSiena manual (Ripley *et al.* 2015). We now describe the modelling process, with emphasis that it is on goodness-of-fit of the model to the data, rather than statistical significance, which is the aim. Fig. S1 is a flow chart illustrating this process.

Convergence

At each step it must first be determined that the model has converged satisfactorily (model verification). This is assessed by convergence t-ratios (one value per parameter; distinct from a t-value which we outline below) and the maximum convergence ratio (one value for the model; both supplied in the RSiena output). The convergence t-ratio for a parameter is the distance between the observed and simulated values of that parameter. Ideally this would be zero, but absolute scores of less than 0.1 indicates the model is robust enough for the result to be fully interpretable, while less than 0.2 are acceptable when first specifying the model (Ripley *et al.* 2015). The maximum convergence ratio is the maximum t-ratio for convergence for any linear combination of the parameters, and by definition is greater than or equal to the maximum t-ratio for single parameters (Snijders 2015). The distance of estimated model terms from true values is better indicated by the maximum convergence ratio than by the t-ratios in isolation (Snijders 2015). Values below 0.25 are considered acceptable (Ripley *et al.* 2015). In SIENA a repeat simulation run can use starting parameter values from the results of a prior run. Using these more accurate initial values should enable better convergence. Starting values from models lacking some parameters of the new model can also be used, e.g. when adding new effects. We highly recommend using initial parameter estimates from simpler models when adding new effects, as in our experience this consistently leads to better convergence in the new model.

Goodness-of-fit

Once the model has converged, the next stage is to test for goodness-of-fit (GOF; model validation). A model that converges is not necessarily a good model to describe the given data. GOF tests allow the researcher to examine whether a model can simulate networks that are structurally similar to the observed networks. Commonly used tests include estimating the degree distribution (number of unique connections each individual possess), the geodesic distribution (the distance in terms of network links an individual is from others in the network), and the triad census (frequency of sets of three individuals possessing zero, one, two or three connections amongst them; Ripley *et al.* 2015; Ilany *et al.* 2015). The results of GOF tests are plotted to assess how the observed values of network statistics compare to simulated values. These plots can help the researcher by suggesting effects to add. We provide examples of both adequate and inadequate GOF plots in figures S2-7. For instance, if the degree distribution plot shows that the model fails to account for the number of isolates in the network (solitary individuals); one can add the *isolates* effect to specifically model this tendency. The Mahalanobis distance (MHD; the distance of the mean of the distribution of the simulated data from the observed value; low values desirable) and the associated test are also used to evaluate GOF. Non-significance indicates the simulated values do not differ from the observed values; see Lospinoso (2012) and the RSiena manual (Ripley *et al.* 2015) for further details. Following Ilany *et al*. (2015), an acceptable GOF is when the p-value for each of the MHDs for degree distribution, geodesic distribution, and the triad census are > 0.05. The statistics for a successful GOF should be reported, either in the supplementary materials or in the main text if it relates to a term relevant to a hypothesis. To achieve proper GOF, we strongly recommend first adding structural effects related to network dynamics before adding terms related to individual or dyadic covariates, or network-trait co-dynamics. In our experience, these are much more likely to increase model GOF than other covariates.

Hypothesis testing

Once the model has satisfactorily converged and possesses acceptable GOF, the researcher can start adding effects relevant to particular hypotheses. Each time an effect is added, repeated runs are performed until the model converges. Following this, the GOF of the new model is examined to determine whether the new term has improved or worsened fit. If the term has worsened fit we recommend it should not be retained for further steps, but if they are relevant to hypotheses terms that do not necessarily improve fit can be kept in the model. Terms with large standard errors (> 4) may also have to be fixed at a particular (large) value, as they can prevent satisfactory convergence (Ripley *et al.* 2015).

Once the model of interest, which converges and demonstrates acceptable GOF, has been arrived at, the estimates and standard errors for effects relevant to particular hypotheses can be evaluated. A simple statistical test can also be carried out: the estimate can be divided by its standard error to give a t-value (not to be confused with the t-ratio used to assess convergence); t-values greater than two indicate significance at 95% (Burk *et al.* 2007). Another option for statistical testing is the score-type test (Ripley *et al.* 2015). This test determines whether the GOF is significantly worsened by constraining the value of the parameter to be zero (for more details see Schweinberger 2012). The score-type test separates the estimation and the testing procedures. This is likely to be preferable in ecological datasets when the model will often have many parameters for the amount of data available (Ripley *et al.* 2015). The convergence t-ratios are ignored for any effect being estimated using a score-type test (Ripley *et al.* 2015). If either t- or score-type tests indicate that an effect could be removed, it is still worth visually assessing the change in GOF, and retaining any terms which do appear to contribute to GOF. It is also good practice to assess whether any effects dropped previously contribute to GOF in the final candidate model. It is possible that terms that did not contribute to GOF alone will improve fit when used in conjunction with other parameters.

Alongside convergence and GOF, the model will also calculate correlations between pairs of parameters. Correlations between parameters are quite likely for network effects, therefore values below 0.9 are acceptable (Ripley *et al.* 2015). Values over 0.9 may mean that one of the effects should not be included, although not in every case (see Snijders *et al* 2010). If two parameters are highly correlated then a model including either of them may achieve good GOF, but will not converge if both are included. In such cases, this should be reported, as it indicates that the two processes the terms represent are linked, which is possibly of biological interest.

Note that in some cases multiple different models will give adequate GOF. These typically will have similar terms, supporting the inclusion of these terms as important in the system of study. There is currently no provision for the direct comparison of different models that is analogous to likelihood ratio tests or the comparison of information criteria in RSiena. In general, we recommend that the simplest model that allows the hypotheses of interest to be investigated should be used. If there appear to be several of these then all should be considered as candidate “best” models, and researchers should discuss why differences in model structure may affect parameter estimates.

## Example

To illustrate how SAOMs are applied, we will now go through an example, using networks of interactions among crickets to investigate how fighting behaviour relates to risk-taking. We provide the data and R code to replicate this example as separate files. As a stochastic process, repeats of the analysis will result in slightly different values. The key qualitative aspects of the result should remain identical however. We note that this dataset does not meet all of our recommendations we set out above (e.g. a large number of individuals are not present for every time period). We however keep the model simple and so satisfactory convergence was achieved. Such a cautious approach may be necessary for those with similar datasets that do not completely fulfil the requirements we described.

Example data

The crickets are field crickets *Gryllus campestris* Linnaeus. The crickets are monitored as part of the WildCrickets project (www.wildcrickets.org), using a network of video cameras to record the behaviours of individually marked, free-living individuals (Rodríguez-Muñoz *et al.* 2010). A short time (3-4 days) after they became adult, and every 10 days subsequently, crickets were trapped, and assayed for risk taking behaviour (a.k.a. boldness: Réale et al 2007) in a laboratory adjacent to the field site (Fisher *et al.* 2015). This consisted of placing a cricket inside an opaque tube, and recording whether it emerged from the tube within 30 minutes. Following a 30-minute rest period in an isolated room, this test was repeated, before the cricket was released back to the burrow it was trapped from. Crickets were scored 0, 1 or 2 at each capture, depending on the number of times they left the tube in the two 30 minute tests they received. Leaving the tube into a potentially dangerous environment represents a risky behaviour, hence higher scores indicate crickets that are bolder.

We were interested in whether boldness was related to fighting behaviour in the field. Crickets engage in (predominantly) intra-sex fights for access to burrows (for protection from predators) and members of the opposite sex (for mating; Alexander 1961). Occasionally crickets of the opposite sex will engage in aggressive interactions. After fighting crickets experience a surge in the neurotransmitter octopamine, which likely contributes to the tendency of individuals that win one fight to win subsequent fights (Rillich & Stevenson 2011; Chang *et al.* 2012). The effect of the neurotransmitter may also carry-over into non-social contexts e.g. risk-taking behaviour (Niemelä & Santostefano 2015). However, as fighting behaviour in the first place is risky, it is also plausible that individuals initially more willing to take risks are then more likely to engage in fights. Finally, it is also possible that there is a latent trait or syndrome of extraversion, which makes crickets both bolder and more aggressive. To investigate how boldness and social interactions relate to each other, we analysed networks of fighting aggregated over eight day periods and scores from the repeated boldness assays in a SAOM.

Of the total population of 239 individuals in 2013, 163 crickets were assayed for boldness and were recorded engaging in at least one fight. We inferred that a pair of crickets who fought at least once in an eight-day period were in a state of “antagonistic relationship”. We constructed three sets of associations among the 163 individuals, spanning a continuous 24-day period. The three networks are illustrated in Fig. S8. These were undirected i.e. symmetrical, so indicated which pairs of crickets fought each other but not who attacked who or who won. This is informative of the extent to which each individual is involved in aggressive interactions, which is relevant to the interests we outline above, rather than position in a dominance hierarchy. Some crickets only became adult part way through this period, or died before the end of it. For these individuals, structural zeroes were inserted into the association matrices for the corresponding rows and columns at the appropriate time points. In RSeina, this is coded as a “10” (presence of relationship as “1”, no relationship as “0”) and structural ones as “11”, although none of the latter were used here.

Along with each of these three association matrices, we entered individuals’ sex as a binary variable. This allowed us to model the tendency for the sexes to differ in the number of fights they have, and for the tendency for fights between a pair to depend on the sex of each of them. Finally, we constructed a 163 x 3 matrix, containing each cricket’s score from its last boldness assay before the start of each of the three time periods. If a cricket was not caught and assayed between two time points then its score from the last time it was assayed was used. This assumes a cricket has an individual specific underlying level of behaviour that may change over time, yet remains consistently different from other individuals, which we have shown using a linear modelling approach (Fisher *et al.* 2015).

Modelling procedure

For this undirected network, we used a forcing type model, where one individual unilaterally decides whether a tie should be formed (i.e. by attacking another cricket) or dissolved (i.e. by leaving the area). Following this, the first step was to model the change of the network over time, without sex or boldness. This initial model by default includes a rate function per time point, giving the rate of change in tie formation between each time point. It also includes a density function, which models the tendency for individuals to be connected to other individuals in the network. This tends to be negative as most social and agonistic networks are sparse (Ripley *et al.* 2015). Finally, the model by default includes the effect of triadic closure, where individuals are more likely to associate if they already share a mutual associate. The parameters of this model converged satisfactorily (convergence t-ratios < |0.2| and maximum convergence ratio < |0.25|). However, the GOF was not satisfactory (outdegree distribution MHD p-value = 0.037, geodesic distribution MHD p-value = 0.048, triad census MHD p-value = 0.13). Visual inspection of the plots indicated that the fit was particularly bad for crickets with many connections. To improve fit we added the effect of indegree popularity. This models the tendency for individuals with many connections to continue having more connections, an indicator of social personality types (Krause, James & Croft 2010; Wilson *et al.* 2012). With this effect the GOF was greatly improved and the MHD p-values for the degree distribution, geodesic distribution and triad census were all > 0.2, indicating that adding indegree popularity resulted in a satisfactory GOF. See Figs. S2-4 for examples of bad GOF for each of the three statistics, and Figs. S5-7 for examples of improved GOF for each of the three statistics. We then gradually built up effects, starting from simple to more complex, examining the effect on GOF for each as it was added.

In our second step, we added two effects simultaneously related to sex: sex on the tendency to form ties and an interaction of sex of the focal individual (ego) and the sex of the potential partner (alter) on tie formation. The former effect models whether the sexes differ in the number of fights they have, while the latter effect models the possibility that individuals of the same sex are much more likely to fight each other than members of the opposite sex. Adding both simultaneously allows us to evaluate whether information about the sex of the individuals improved the fit of the model to the data, but independent estimates of the effect of each parameter are provided. This model did not initially satisfactorily converge, so we re-ran the model, using results of the initial run as starting values for the parameters as recommended by Ripley *et al.* (2015). This model converged adequately, so we inspected the GOF plots. GOF was not worsened by including these terms, so they were retained. The effect of sex alone appeared quite weak, based on the size of the effect and its standard error, giving a non-significant t-value. However, the choice of effects in the final model should not be based on significance, but rather on model fit as described above. If an effect does not contribute to convergence and GOF and is not relevant to a particular hypothesis we recommend removing it to obtain simpler models with smaller number of parameters. Effects relevant to a particular hypothesis should be retained to the final model however if they do not prevent convergence or worsen GOF.

Finally, we added the behavioural covariate for boldness, and jointly added the effects of number of connections (degree) on boldness score at the next time point, and boldness score on the number of connections at the next time point. Either process could lead to an association between boldness and number of connections, but simultaneously estimating them will indicate which, if either, is influential in the cricket network. When a behavioural covariate is added, some default effects are automatically added. These are rate functions for each time point as for the network process, the linear change of the behaviour over time, and the quadratic change in the behaviour over time. The linear change is simply the change in absolute value of the behaviour over time i.e. do crickets get more/less bold with age. The quadratic change models the effect of an individual’s behaviour on itself i.e. is there a positive or negative feedback loop. A positive value indicates behaviour is self-reinforcing (e.g. addictive behaviour), while a negative value suggests regression to the mean (Snijders *et al.* 2010). The model converged adequately, so we inspected the GOF plots for the behavioural variable and the other network statistics. These were satisfactory, suggesting we had arrived at our final model to test our hypotheses, and could continue with interpretation of its results.

Model summary

Final model results, with effect sizes, standard errors, t-values and convergence t-ratios are presented in Table S1. This is how previous studies have reported results from SAOMs (e.g. Burk, Steglich & Snijders 2007), so we recommend others present their results in this way. The t-values for sex, degree on behaviour and behaviour on degree were all < 2, indicating they do not have a significant effect. The effects of density, indegree popularity, triadic closure, the interaction between sex of the ego and sex of the alter and both the linear and quadratic changes in risk-taking behaviour all possessed t-values > 2. This was also confirmed by score-type tests for each parameter. Therefore, we have demonstrated that: 1) crickets are more likely to fight a particular individual if both have fought a third cricket; 2) crickets that are involved in fights with more conspecifics tend to fight even more individuals at the next time step; 3) the sexes are involved in fights at equal frequency; 4) fights strongly tend to be with members of the same sex; 5) crickets’ boldness increases over time; 6) laboratory individuals’ boldness levels get relatively more different from each other with time and 7) a cricket’s social interactions are not related to its boldness in the lab. Findings 5 & 6 have previously been found using a linear modelling approach (Fisher *et al.* 2015). In fact, all these findings may have been obtainable through multiple separate sets of analysis, but with a SAOM they can be simultaneously evaluated in one framework. This allows the influence of the effects to be accounted for when estimating the others, which should decrease the chance of type 1 statistical errors.

Table S1. Results of full model, to three significant figures. The t-statistic is the estimate divided by the standard error, values greater than |2| are considered statistically significant at the 95% level. Convergence t-ratios are used to assess the fit of the model to the data, with values <|0.1| required to publish results. The “Interpretation” column refers to which bullet point in the Model summary section the effect relates to.

| Parameter | Estimate | Standard error | T-statistic | Convergence t-ratio | Interpretation |
| --- | --- | --- | --- | --- | --- |
| Network rate 1 | 1.22 | 0.192 | 6.38 | NA | NA |
| Network rate 2 | 1.28 | 0.170 | 7.50 | NA | NA |
| Density | -3.95 | 0.332 | -11.9 | -0.054 | NA |
| Transitive triads | 1.128 | 0.406 | 2.79 | 0.000 | 1 |
| Indegree popularity | 0.187 | 0.071 | 2.64 | -0.044 | 2 |
| Sex | -0.049 | 0.224 | -0.222 | -0.038 | 3 |
| Sex ego – sex alter interaction | 5.450 | 1.107 | 4.92 | -0.045 | 4 |
| Behaviour rate 1 | 1.60 | 0.427 | 3.74 | 0.019 | NA |
| Behaviour rate 2 | 1.15 | 0.275 | 4.20 | -0.052 | NA |
| Behaviour linear effect | 0.606 | 0.192 | 3.15 | 0.051 | 5 |
| Behaviour quadratic effect | 0.894 | 0.216 | 4.14 | -0.099 | 6 |
| Degree on behaviour | -0.019 | 0.126 | -0.149 | 0.081 | 7 |
| Behaviour on degree | -0.0004 | 0.300 | -0.001 | 0.004 | 7 |

Maximum convergence t-ratio = 0.158

## References

Alexander, R.D. (1961) Aggressiveness, territoriality, and sexual behavior in field crickets (Orthoptera: Gryllidae). *Behaviour*, **17**, 130–223.

Aplin, L.M., Farine, D.R., Morand-Ferron, J., Cockburn, A., Thornton, A. & Sheldon, B.C. (2015) Experimentally induced innovations lead to persistent culture via conformity in wild birds. *Nature*, **518**, 538–541.

Burk, W.J., Steglich, C.E.G. & Snijders, T.A.B. (2007) Beyond dyadic interdependence: Actor-oriented models for co-evolving social networks and individual behaviors. *International Journal of Behavioral Development*, **31**, 397–404.

Chang, C., Li, C.-Y., Earley, R.L. & Hsu, Y. (2012) Aggression and related behavioral traits: the impact of winning and losing and the role of hormones. *Integrative and comparative biology*, **52**, 801–13.

Fisher, D.N., David, M., Tregenza, T. & Rodriguez-Munoz, R. (2015) Dynamics of among-individual behavioral variation over adult lifespan in a wild insect. *Behavioral Ecology*, **26**, 975–985.

Frère, C.H., Krützen, M., Mann, J., Watson-Capps, J.J., Tsai, Y.J., Patterson, E.M., Connor, R., Bejder, L. & Sherwin, W.B. (2010) Home range overlap, matrilineal and biparental kinship drive female associations in bottlenose dolphins. *Animal Behaviour*, **80**, 481–486.

Hipp, J.R., Wang, C., Butts, C.T., Jose, R. & Lakon, C.M. (2015) Research Note: The consequences of different methods for handling missing network data in Stochastic Actor Based Models. *Social networks*, **41**, 56–71.

Huisman, M. & Steglich, C. (2008) Treatment of non-response in longitudinal network studies. *Social Networks*, **30**, 297–308.

Ilany, A., Booms, A.S. & Holekamp, K.E. (2015) Topological effects of network structure on long-term social network dynamics in a wild mammal. *Ecology Letters*, **18**, 687–695.

Jaccard, P. (1901) Étude comparative de la distribution florale dans une portion des Alpes et des Jura. *Bulletin de la Société Vaudoise des Sciences Naturelles*, **37**, 547–579.

Krause, J., James, R. & Croft, D.P. (2010) Personality in the context of social networks. *Philosophical transactions of the Royal Society of London. Series B, Biological sciences*, **365**, 4099–4106.

Lospinoso, J.A. (2012) *Statistical Models for Social Network Dynamics*. University of Oxford.

Niemelä, P.T. & Santostefano, F. (2015) Social carry-over effects on non-social behavioral variation: mechanisms and consequences. *Frontiers in Ecology and Evolution*, **3**, 49.

Réale, D., Reader, S.M., Sol, D., McDougall, P.T. & Dingemanse, N.J. (2007) Integrating animal temperament within ecology and evolution. *Biological Reviews*, **82**, 291–318.

Rillich, J. & Stevenson, P.A. (2011) Winning fights induces hyperaggression via the action of the biogenic amine octopamine in crickets. (ed M Briffa). *PloS one*, **6**, e28891.

Ripley, R.M., Snijders, T.A.B., Boda, Z., Voros, A. & Preciado, P. (2015) Manual for SIENA version 4.0 (version October 10, 2015).

Rodríguez-Muñoz, R., Bretman, A., Slate, J., Walling, C.A. & Tregenza, T. (2010) Natural and sexual selection in a wild insect population. *Science*, **328**, 1269–1272.

Schweinberger, M. (2012) Statistical modelling of network panel data: goodness of fit. *The British Journal of Mathematical and Statistical Psychology*, **65**, 263–281.

Snijders, T.A.B. (2015) Siena Advanced Users’ Workshop.

Snijders, T. a. B., van de Bunt, G.G. & Steglich, C.E.G. (2010) Introduction to stochastic actor-based models for network dynamics. *Social Networks*, **32**, 44–60.

Wang, J. (2007) *Simulation Studies of Power and Robustness in Models for Network Dynamics*. University of Oxford.

Wang, C., Butts, C.T., Hipp, J.R., Jose, R. & Lakon, C.M. (2016) Multiple Imputation for Missing Edge Data: A Predictive Evaluation Method with Application to Add Health. *Social networks*, **45**, 89–98.

Wilson, A.D.M., Krause, S., Dingemanse, N.J. & Krause, J. (2012) Network position: a key component in the characterization of social personality types. *Behavioral Ecology and Sociobiology*, **67**, 163–173.

## Figures

Figure S1. A flow chart to illustrate the modelling process. Blue indicates actions, while green and red distinguish between results that might lead to term retention or removal respectively. Note the focus is on achieving an adequate goodness-of-fit (GOF), with the statistical significance of terms only considered at the penultimate step. In some cases following this process will result with multiple models with adequate GOF. This will typically support the inclusion of particular effects that are present in both models. If two parameters are highly correlated then a model including either of them may achieve good GOF, but will not convergence if both are included.

Figures S2-4. Bad goodness-of-of fit for the statistic outdegree distribution (the number of other crickets and individual is connected to) the geodesic distribution (number of steps through the network an individual is form another other) and the triad census (of all sets of three crickets, the frequency there are 0 (003), 1 (102), 2 (201) or 3 (300) connections among them). The simulated values are black dots (the median), grey boxes (the first and third quartiles), crosses (outliers) and the smooth lines surrounding (approximating to the density of the statistic). These show a poor match with the observed values in the networks (red points and line). Wavvy density estimates indicate that the simulation estimates a few discrete values, usually integers. All statistics have been mean centred and set to the same scale, to allow easier comparison of fit for different values of each statistic.

Figures S5-7. Improved goodness-of-of fit for the statistic outdegree distribution (the number of other crickets and individual is connected to) the geodesic distribution (number of steps through the network an individual is form another other) and the triad census (of all sets of three crickets, the frequency there are 0 (003), 1 (102), 2 (201) or 3 (300) connections among them). The simulated values are black dots (the median), grey boxes (the first and third quartiles), crosses (outliers) and the smooth lines surrounding (approximating to the density of the statistic). These an improved match with the observed values in the networks (red points and line), with the red points typically falling within the grey box and near to the median simulation score. Wavvy density estimates indicate that the simulation estimates a few discrete values, usually integers. All statistics have been mean centred and set to the same scale, to allow easier comparison of fit for different values of each statistic.

Figure S8. Social network of *Gryllus campestris* over three time periods. Each time period comprises of eight days. Associations are based on two crickets engaging in an antagonistic interaction. Square symbols indicate males, circles females. Individuals retain the same position in each time period, based on their centrality across the entire field season. If the individual was were either not an adult yet or dead in a time period they are not plotted.
